# Host hydrocarbons protect symbiont transmission from a radical host defense

**DOI:** 10.1073/pnas.2302721120

**Published:** 2023-07-24

**Authors:** Chantal Selina Ingham, Tobias Engl, Bernal Matarrita-Carranza, Paul Vogler, Bruno Huettel, Natalie Wielsch, Aleš Svatoš, Martin Kaltenpoth

**Affiliations:** ^a^Department of Evolutionary Ecology, Institute of Organismic and Molecular Evolution, Johannes Gutenberg-University Mainz, Mainz 55128, Germany; ^b^Department of Insect Symbiosis, Max-Planck-Institute for Chemical Ecology, Jena 07745, Germany; ^c^Max Planck Genome Centre Cologne, Max Planck Institute for Plant Breeding Research, Cologne 50829, Germany; ^d^Research Group Mass Spectrometry/Proteomics, Max-Planck-Institute for Chemical Ecology, Jena 07745, Germany

**Keywords:** nitric oxide, defensive symbiosis, *Streptomyces*, mutualism, immune system

## Abstract

Symbioses with microbes play a pivotal role in the evolutionary success of insects, and can lead to intimate host–symbiont associations. However, how the host maintains a stable symbiosis with its beneficial partners while keeping antagonistic microbes in check remains incompletely understood. Here, we uncover a mechanism by which a host protects its symbiont from the host’s own broad-range antimicrobial defense during transmission. Beewolves, a group of solitary digger wasps (Hymenoptera: Crabronidae), provide their brood cells with symbiotic *Streptomyces* bacteria that are later transferred to the cocoon and protect the offspring from opportunistic pathogens by producing antibiotics. In the brood cell, however, the symbiont-containing secretion is exposed to a toxic burst of nitric oxide (NO) released by the beewolf egg, which effectively kills antagonistic microorganisms. How the symbiont survives this lethal NO burst remained unknown. Here, we report that upon NO exposure in vitro, the symbionts mount a global stress response, but this is insufficient to ensure survival at brood cell-level NO concentrations. Instead, in vivo bioassays demonstrate that the host’s antennal gland secretion (AGS) surrounding the symbionts in the brood cell provides an effective diffusion barrier against NO. This physicochemical protection can be reconstituted in vitro by beewolf hydrocarbon extracts and synthetic hydrocarbons, indicating that the host-derived long-chain alkenes and alkanes in the AGS are responsible for shielding the symbionts from NO. Our results reveal how host adaptations can protect a symbiont from host-generated oxidative and nitrosative stress during transmission, thereby efficiently balancing pathogen defense and mutualism maintenance.

## Significance

Periods of exposure to environmental pressures during transmission across host generations are universal to extracellular symbionts of many Hemiptera, Coleoptera, some Hymenoptera and Diptera, as well as other insect orders. Given that vertically transmitted symbionts generally exhibit signatures of genome erosion and metabolic streamlining, host adaptations to protect the symbiont from abiotic and biotic challenges during transmission should be widespread, but remain poorly studied to date. Here, we show that a beewolf host embalms its antibiotic-producing “*Streptomyces philanthi*” symbionts in a secretion containing long-chain hydrocarbons, which effectively blocks diffusion of toxic nitric oxide produced by the beewolf egg. This host adaptation protects the symbionts from nitrosative and oxidative stress during the vulnerable period of extracellular transmission.

Microbial symbioses are ubiquitous in nature, and they constitute important drivers of evolutionary innovation (1, 2). In insects, symbionts confer a wide range of benefits to their hosts, such as nutrient supplementation, digestion, detoxification, and protection from predators, pathogens, and parasites (3–5), enabling their hosts to colonize otherwise inaccessible niches (3). As a symbiont establishes a persistent beneficial association with the host insect, it must overcome the challenges posed by the host-associated lifestyle (6). This includes, but is not limited to, coping with host physiology–related stressors, such as host immune responses (7–13), high levels of reactive oxygen species (14, 15), as well as dealing with unfavorable conditions outside the host in case of external transmission (16). While symbiont factors to cope with host immune effectors have been intensively studied (7, 17–24), much less is known about host adaptations that prevent exposure or protect the symbionts from the host’s own immune effectors as well as environmental stressors (but see refs. 7, 13, 14, and 25–28).

A common stressor for symbionts is the exposure to reactive oxygen or nitrogen species (ROS and RNS, respectively). In particular, nitric oxide (NO) is an important effector molecule in both mutualistic and pathogenic interactions (29, 30), but also serves as a signaling molecule at low concentrations (reviewed e.g. in refs. 31 and 32). A regulatory function in establishing mutualistic interactions with microorganisms has been recognized in the squid–*Aliivibrio* symbiosis, where NO is essential in symbiosis establishment and mediates host-symbiont specificity (33, 34). In the legume–rhizobium interaction, it contributes to the regulation of nodule formation (35), whereas in the coral–dinoflagellate symbiosis stressful climatic conditions lead to high levels of host-produced NO, resulting in the breakdown of symbiosis (36). As a constituent of the immune system (reviewed e.g. in refs. 29 and 32), NO is produced by vertebrate macrophages (29) and by insect hemocytes, fat body and midgut cells to combat pathogens (32). Its cytotoxic effects stem from its diffusion through cell membranes (32) and the formation of highly reactive radicals upon reaction with oxygen or super- and peroxides (37), which in turn inflict oxidative damage upon macromolecules, such as DNA, RNA, proteins, lipids, and prosthetic groups (37, 38).

**Fig. 1.**
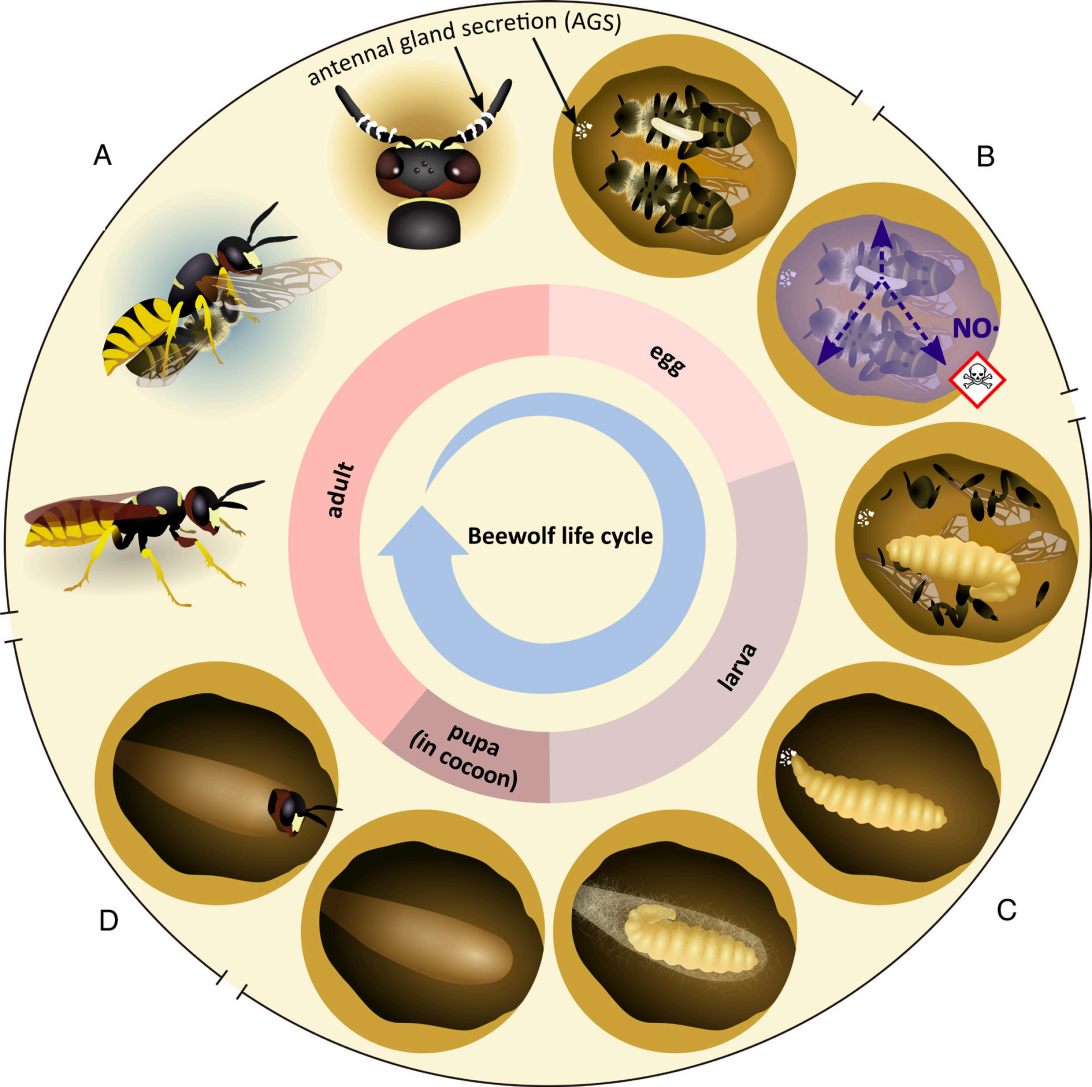
Life cycle of the European Beewolf (*P. triangulum*). (*A*) Beewolf females hunt *Apis mellifera* workers and paralyze them by injecting their venom into the bee’s thorax (51). They provide their subterranean brood cells with one to several bees, embalmed in a secretion from their postpharyngeal gland (41). This secretion contains high amounts of long-chain saturated and unsaturated hydrocarbons that prevent infection of the provisions by reducing water condensation (41–44). The female deposits an egg on top of the provisions (52). Before sealing the brood cell, the female applies an AGS rich in linear unsaturated and saturated hydrocarbons (53) and containing the defensive symbiont *Streptomyces philanthi* to the brood cell ceiling (46, 54). (*B*) The beewolf egg sanitizes the brood cell by releasing high amounts of toxic nitric oxide (NO), with NO emission peaking at ~14 to 16 h after oviposition (39). While NO effectively kills microbial opportunists in the brood cell (39), *S. philanthi* withstands NO via a previously unknown mechanism. (*C*) After hatching and feeding on the provisioned bees for several days, the larva integrates *S. philanthi* into its cocoon (46). On the cocoon surface, the symbionts produce an antibiotic cocktail (47, 55) that provides protection from microbial infestation (46, 47). (*D*) After 4 to 6 wk or in the following summer, the larva undergoes metamorphosis, and the adult ecloses from the cocoon (52, 56).

Recently, an intriguing case of NO as an external immune defense has been described in beewolf wasps (Hymenoptera: Crabronidae) (39). These solitary digger wasps construct subterranean brood cells (Fig. 1), where the offspring is confronted with a diverse community of opportunistic mold fungi during development (40). In response, beewolves have evolved multiple adaptations to prevent infections. First, the eggs of the European Beewolf (*Philanthus triangulum*) fumigate the brood cell with high concentrations of NO (1,690 ± 680 ppm), effectively killing antagonistic soil-dwelling microbes (Fig. 1) (39). Second, female beewolves embalm the larval provisions in a secretion from their postpharyngeal gland containing high amounts of long-chain saturated and unsaturated hydrocarbons that prevent infection of the provisions by reducing water condensation and thereby impairing germination of mold fungi (41–44). Last, female beewolves harbor symbiotic “*Streptomyces philanthi*” bacteria in specialized antennal reservoirs (45) and secrete them into the brood cell (46). Later, the larva incorporates the deposited symbionts into its cocoon during spinning (46). On the cocoon, the symbionts produce an antibiotic cocktail containing several piericidin and streptochlorin derivatives (47) that effectively protect the offspring from pathogen infestation during the vulnerable phases of hibernation and metamorphosis (46). The symbiosis between beewolves and *Streptomyces* originated about 68 Mya (48), with the symbionts providing a long-term stable combination prophylaxis (49) despite signatures of ongoing erosion in the *P. triangulum* symbiont genome (50). Interestingly, because female beewolves secrete *S. philanthi* into the brood cell prior to NO fumigation, the symbionts are exposed to the high concentrations of NO while they are embedded in the antennal gland secretion (AGS). However, the mechanisms allowing the symbionts to survive NO exposure remain enigmatic.

Here, we set out to elucidate whether host or symbiont adaptations are responsible for protecting the symbionts from recurrent NO exposure in the beewolf brood cell. We first assessed *S. philanthi* survival upon ecologically relevant NO concentrations in vitro, in comparison to the free-living relative *Streptomyces coelicolor*. We then used RNAseq and proteomics to characterize the symbiont’s response to NO exposure in vitro and in vivo. Additionally, we examined the potential for host-mediated protection of the symbionts by screening the AGS proteome for proteins with putative protective functions against NO, and by testing the capacity of the AGS to act as an NO diffusion barrier. Our results reveal that the symbionts up-regulate protective enzymes upon NO exposure, but these are insufficient to protect the bacteria from the high NO concentration in the brood cell. However, the AGS provides efficient protection from NO, an effect that can be reconstituted in vitro by a beewolf hydrocarbon extract and by synthetic (Z)-9-tricosene. Our findings uncover a physicochemical mechanism that a host uses to protect its symbiont from oxidative and nitrosative stress during the vulnerable period outside of the host environment, thereby stabilizing the symbiotic association and ensuring symbiotic benefits during hibernation and metamorphosis.

## Results

### Symbionts Mount a Global Stress Response but Are Susceptible to NO In Vitro.

We first sought to find out whether symbionts show resistance to NO in vitro. We investigated the effect of NO at brood cell-level concentration (10× injection of 1 mL 1% NO in N_2_ vs. pure N_2_ into the headspace of the culture containers) on “*S. philanthi* biovar *triangulum*” strain 23Af2 of *P. triangulum* (henceforth *S. philanthi*) and the free-living *Streptomyces coelicolor* M145 A2(3) (henceforth *S. coelicolor*). A qualitative survival assessment with a fluorescent live-dead stain indicated that NO drastically reduced the survival of both species at all timepoints (*SI Appendix*, Fig. S1).

We then set out to characterize the molecular response of both species to NO. To this end, we exposed *S. philanthi* and *S. coelicolor* to 1% NO in N_2_ again, but this time analyzed the respective changes in gene expression 2 h and 6 h post exposure in comparison to an N_2_ control treatment. Additionally, we examined the NO response of *S. philanthi* in vivo by analyzing the gene expression of symbionts in the AGS in the presence and absence of the NO-emitting egg. Genes with at least twofold and significant expression change were considered differentially expressed in all datasets.

**Fig. 2.**
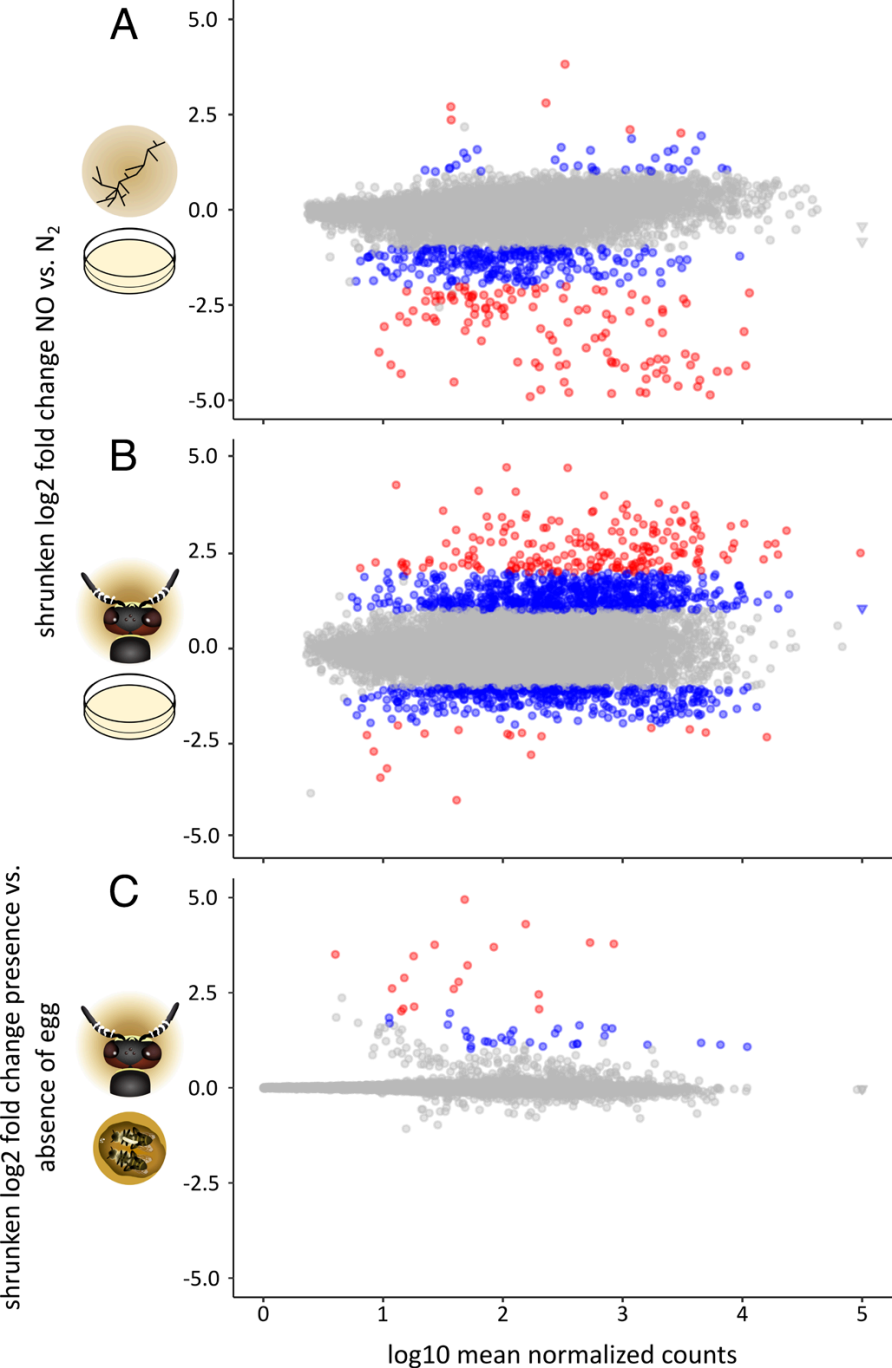
Changes in gene expression in (*A*) free-living *S. coelicolor* (indicated by the symbol of filamentous bacteria) and (*B*) symbiotic *S. philanthi* (indicated by the beewolf head) 6 h after in vitro exposure to NO in comparison to exposure to N_2_ (indicated by the petri dish), and in (*C*) symbiotic *S. philanthi* in the AGS within beewolf brood cells in the presence vs. the absence of a beewolf egg emitting NO (indicated by the beewolf brood cell). Significant gene expression differences are highlighted in color (adjusted *P* < 0.05 and twofold to fourfold differential expression in blue, and more than fourfold change in red). Log-transformed values of some extremely highly expressed genes were set to 5 to improve readability and are indicated with triangles instead of circles.

For both symbiotic *S. philanthi* and free-living *S. coelicolor*, most of the genes encoded in their genomes were found to be expressed across all conditions, with the replicates showing consistent gene expression profiles (*SI Appendix*, Tables S1 and S2 and Fig. S2). *S. coelicolor* showed a moderate response to NO, with 353 differentially expressed genes after 2 h, and 413 after 6 h (Fig. 2*A* and *SI Appendix*, Fig. S3*A*). Similarly, *S. philanthi* gene expression was not profoundly affected by NO after 2 h. At this timepoint, 218 up- and 37 down-regulated genes accounted for a total of 255 differentially expressed genes (*SI Appendix*, Figs. S3*B* and S4). However, we found a drastic change in *S. philanthi* gene expression 6 h after NO exposure (Fig. 2*B*). 895 up- and 460 down-regulated genes accounted for a total of 1,355 differentially expressed genes (*SI Appendix*, Fig. S4). Strikingly, *S. philanthi* exposed to NO released by the beewolf egg in vivo did not show a strong response to NO, with only 50 genes being differentially expressed, all of them up-regulated (Fig. 2*C* and *SI Appendix*, Fig. S4*A*).

In addition to the global changes in gene expression profiles, we also assessed the expression of genes known to be involved in bacterial responses to NO (reviewed in ref. 57 and *SI Appendix*, Fig. S5). The flavohemoprotein Hmp converts NO to nitrate (58), and superoxide dismutases convert superoxide anions to hydrogen peroxide (59, 60). The latter is subsequently converted to water by catalases (61, 62). Low-molecular-weight thiols, such as mycothiol and cysteine, serve as antioxidants (57), and small oxidoreductases such as thioredoxin reduce protein thiols (63). Protein chaperones reconstitute the native conformation of oxidized proteins (64), and DNA-binding proteins deflect oxidative damage from the DNA (65). Cellular levels of free ferrous iron are kept low to avert additional oxidative stress caused by ferrous iron via Fenton chemistry (66, 67). At the same time, iron–sulfur clusters, which are essential components of a multitude of proteins (68) but damaged by RNS and ROS, are replenished (69).

**Fig. 3.**
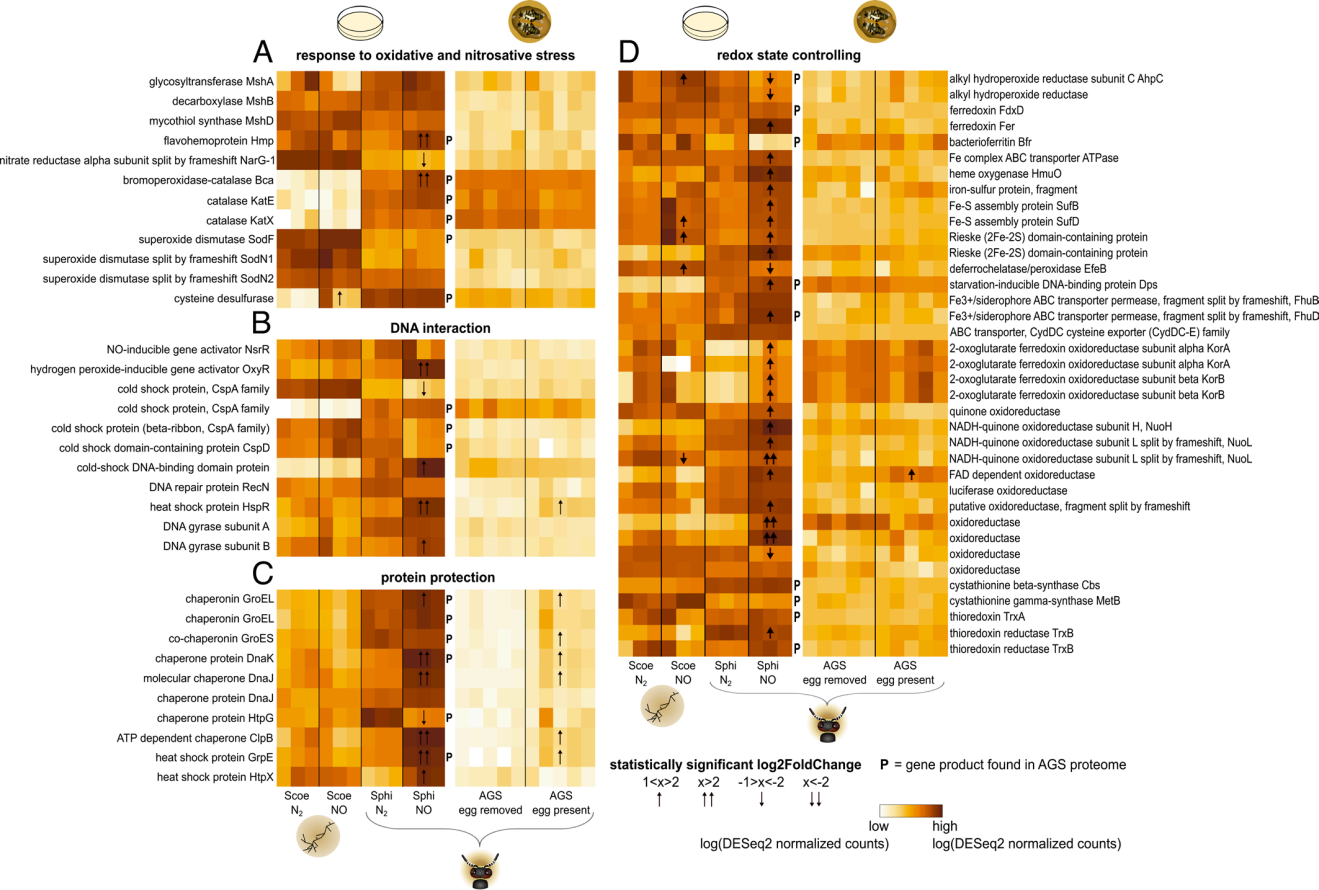
Symbionts display a drastic change in the expression of general stress response genes, as well as oxidative and nitrosative stress–specific responses in vitro, but not in vivo (i.e., within the beewolf brood cell). Treatment-specific expression of genes associated with (*A*) the response to nitrosative and oxidative stress, (*B*) DNA interaction, (*C*) protein protection, and (*D*) redox state controlling. Scoe = free-living *S. coelicolor* (also indicated by the symbol of filamentous bacteria), Sphi = symbiotic *S. philanthi* (also indicated by the beewolf head), AGS = antennal gland secretion. The petri dish indicates gene expression in vitro, the beewolf brood cell symbolizes gene expression within the AGS. The *P* denotes genes for which the respective protein was detected in the proteomic analysis of the AGS.

We identified 69 genes in the *S. philanthi* genome involved in these pathways, as well as their homologues in *S. coelicolor* (Fig. 3). In *S. coelicolor*, we found a relatively high constitutive expression of flavohemoprotein, superoxide dismutases, enzymes of the mycothiol biosynthesis pathway, cold shock proteins, oxidoreductases, and genes related to iron homeostasis, such as ferredoxins (Fig. 3). However, only a few genes belonging to redox and iron homeostasis pathways were differentially expressed after NO exposure in vitro (Fig. 3). By contrast, in *S. philanthi*, most NO stress–related genes were up-regulated after NO exposure, especially those involved in redox and iron homeostasis (Fig. 3). Additionally, many nondifferentially expressed genes exhibited high constitutive expression levels. In stark contrast, only a small number of genes, mainly encoding chaperones, were up-regulated in *S. philanthi* after in vivo exposure to the beewolf egg (Fig. 3). In addition, 20 genes without a corresponding homologue (18 for *S. philanthi*, 2 for *S. coelicolor*) exhibited differential expression after NO exposure (*SI Appendix*, Fig. S6), including genes coding for proteins involved in iron uptake in *S. philanthi*.

### The AGS Contains Host- and Symbiont-Derived Proteins with Protective Properties against NO.

Many of the proteins involved in protection against oxidative and nitrosative damage were also detected in our proteomic analysis of the AGS, including several chaperones, catalases, superoxide dismutase, flavohemoprotein, and many genes involved in redox state controlling (Fig. 3, *SI Appendix*, Fig. S7, and Dataset S1). In addition to these symbiont-produced proteins, the proteome analysis also revealed several host factors antagonizing oxidative and nitrosative stress. Apart from a superoxide dismutase and a heat shock protein of insect origin, we also identified a glutathione synthase, a glutathione S-transferase and a glutathione peroxidase (70).

### NO Exposure Does Not Influence Antibiotic Production by the Symbionts In Vivo.

We tested whether the NO release by the beewolf egg influenced the symbiont-mediated defense of the larva, as NO serves as a signal affecting morphological differentiations and antibiotic production in free-living *Streptomyces* (71–73). Specifically, we examined the impact of NO on symbiont titer and antibiotic production on the cocoon in a semi-natural setting (*SI Appendix*, Fig. S8). However, neither the symbiont titer nor the amount of the three major antibiotics, i.e. piericidin A1 and B1 and streptochlorin on the cocoon was influenced by prior NO exposure of the symbionts in the brood cell (*SI Appendix*, Fig. S8 and Tables S3–S5; symbiont titer: paired *t* test, t = −0.835, df = 8, *P*-value = 0.428, N = 9; antibiotic production: paired *t* tests, *P* > 0.05, N = 5).

### AGS Provides Protection against NO In Vivo.

**Fig. 4.**
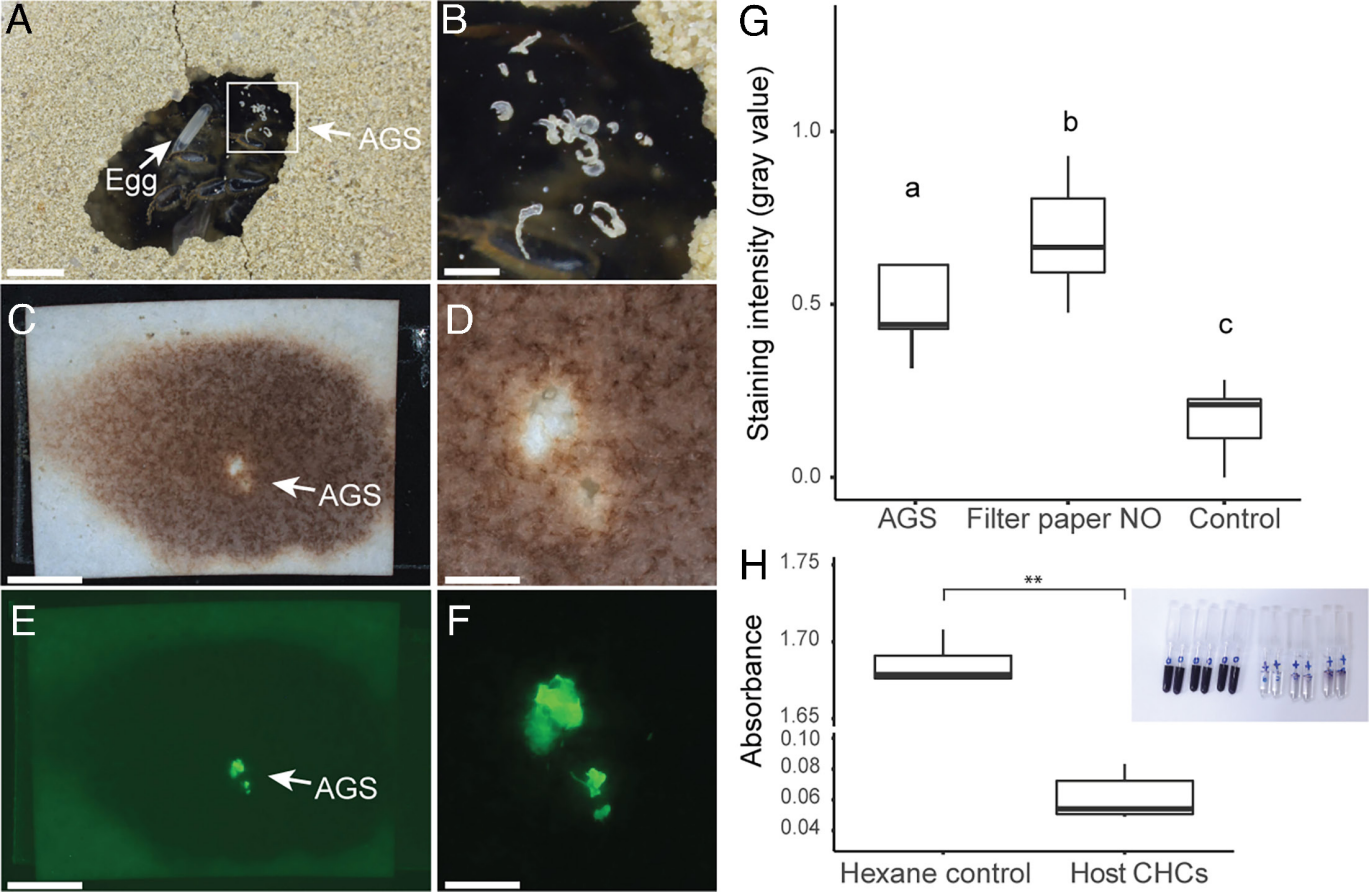
The beewolf female’s AGS blocks diffusion of NO both in vivo and in vitro. (*A*) Beewolf brood cell in an observation cage, with paralyzed bees at the bottom of the brood cell and the beewolf egg on top of one of the bees. The AGS is visible as small white specks at the ceiling of the brood cell, in this case a transparent plastic sheet. (*B*) Enlarged region of the brood cell from *A*, showing the AGS on the brood cell ceiling. (*C*) Representative filter paper prepared with NO indicator that turned brown after NO exposure in the brood cell, with a clear zone where the AGS is localized, enlarged in (*D*). (*E* and *F*) Same areas as in *C* and *(D*) under fluorescent light, corroborating the position of the AGS by its autofluorescence. [Scale bars: (*A*, *C*, and *E*): 4 mm; (*B*, *D*, and *F*): 1 mm.] (*G*) Quantification of indicator reaction upon NO exposure in beewolf brood cells. Comparison of normalized mean gray values between zones of filter paper treated with AGS vs. the surrounding area exposed to NO in the brood cell, and a control without NO exposure (artificial brood cell without beewolf egg that was excavated next to the target brood cell). Different letters above the boxes indicate significant differences (Tukey’s HSD, *P* < 0.05). (*H*) Quantification of indicator reaction upon NO exposure in solutions with a cover of beewolf CHCs vs. a control of hexane (Wilcoxon rank sum exact test, W = 0, *P* = 0.002, N = 6). The *Inset* shows the dark blue indicator solution in control tubes after NO exposure (*Left*) as compared to the transparent solutions covered by beewolf CHCs that prevent NO diffusion (*Right*).

Considering that the symbionts do not survive ecologically relevant concentrations of NO in vitro, we examined whether the host-secreted AGS has the potential to protect *S. philanthi* from NO exposure in the beewolf brood cell (Fig. 4 *A* and *B*). We used the change in coloration of a potassium–iodide–starch oxidation indicator to visualize NO exposure in situ. We transferred the AGS on a filter paper impregnated with the indicator solution, and applied it across the ceiling of the brood cell during NO production by the beewolf egg. We found that the presence of the AGS prevented the color change of the filter paper upon NO release by the beewolf egg (Fig. 4 *C* and *D*). We made use of AGS autofluorescence to confirm its location on the specific noncolored zone of the indicator filter paper (Fig. 4 *E* and *F*). A comparison of the mean gray values, i.e. the mean darkness of the respective pixels, confirmed that the change in coloration of this noncolored, AGS-bearing zone was strongly and significantly reduced compared to the surrounding area that was not covered by AGS (Fig. 4*G*; Tukey’s HSD, *P* < 0.05, N = 9). However, the AGS-bearing zone still displayed a slightly darker hue than a control filter paper without NO exposure (Fig. 4 *C* and *G* and *SI Appendix*, Table S6 and Fig. S9; Tukey’s HSD, *P* < 0.05, N = 9), indicating that low amounts of NO can either penetrate the AGS or diffuse through the filter paper from the sides.

### Beewolf Hydrocarbon Extracts Provide an NO Diffusion Barrier In Vitro.

The AGS is mainly composed of unsaturated and saturated hydrocarbons (53), hence we considered hydrocarbons the primary candidates responsible for the observed protection from NO exposure. To test this, we extracted beewolf cuticular hydrocarbons (CHCs) from two adult males per extract in hexane. The beewolf CHC composition was previously shown to correspond to that of the AGS in both composition and relative amounts of hydrocarbons (53, 74, 75). As beewolf males produce copious amounts of a CHC-derived sex pheromone in specialized head glands, we removed the head during extraction, resulting in an extract of CHCs from thorax and abdomen. We applied the CHCs as a layer on top of 40 µL of an NO indicator iodide–starch solution in qPCR tubes, and exposed the tubes to synthetic NO (0.01% in N_2_). After 1 h, the hexane-treated control solutions had turned dark blue due to the exposure to NO, while the solutions covered with beewolf CHCs remained transparent, similar to control solutions that were not exposed to NO. Concordantly, spectrophotometric analysis revealed a clear inhibition of oxidation (i.e. less change in coloration) of the indicator iodide–starch solution in the samples covered with beewolf CHCs as compared to hexane-treated controls (Fig. 4*E* and *SI Appendix*, Table S7, Wilcoxon rank sum exact test, W = 0, *P* = 0.002, N = 6).

### Synthetic (Z)-9-tricosene Rescues *S. philanthi* Survival upon NO Exposure In Vitro.

To directly confirm the activity of hydrocarbons to protect the symbionts from NO, we covered *S. philanthi* with (Z)-9-tricosene and exposed it to a lethal NO concentration. (Z)-9-tricosene is a constituent of the AGS. It is liquid at room temperature and applicable to the symbionts without a harmful solvent. We monitored macroscopically visible growth for 8 d. (Z)-9-tricosene rescued *S. philanthi* growth in all five replicates, while control inoculants without (Z)-9-tricosene did not visibly grow (*SI Appendix*, Fig. S10).

### *S. philanthi* Cells Are Embedded within the AGS Matrix In Vivo.

We performed scanning electron microscopy on the AGS to assess whether the symbiont cells are indeed embedded within the AGS matrix. While symbiont cells growing in vitro showed the typical mycelial structure (*SI Appendix*, Fig. S11 *A* and *B*), the AGS exhibited a smooth surface, with symbiont cells being either not visible at all or appearing covered by a film of AGS (*SI Appendix*, Fig. S11 *C*–*F*). Thus, most or all of the symbionts are likely shielded by the hydrocarbon-rich AGS from the external environment.

## Discussion

During transmission, the beewolf’s defensive symbiont-bearing secretion experiences toxic nitric oxide (NO) concentrations released by the beewolf egg for brood cell sanitation. The mechanisms ensuring *S. philanthi* survival and therefore the long-term stability of the symbiosis remained enigmatic. Here, we show that *S. philanthi* mounts a profound stress response upon NO exposure in vitro, but the majority of cells do not withstand NO concentrations resembling those in the brood cell. However, our in vivo assays revealed that the host’s symbiont-bearing AGS serves as an efficient diffusion barrier against NO, an effect that we could reconstitute in vitro using beewolf cuticular hydrocarbon (CHC) extracts with a composition representing the hydrocarbon profile in the AGS, as well as using a single synthetic hydrocarbon to rescue *S. philanthi* survival upon otherwise lethal NO stress.

### Symbiont Response to NO Exposure.

Surprisingly, in vitro exposure to NO in ecologically relevant concentrations was not only lethal to the free-living soil bacterium *S. coelicolor*, but also killed most cells of the beewolf symbiont *S. philanthi*. As opposed to *S. coelicolor,* however, *S. philanthi* displayed major gene expression changes upon NO exposure, particularly characterized by the upregulation of genes involved in scavenging NO and its reaction products as well as those preventing or alleviating cellular damage inflicted by RNS and ROS. Microbial responses to protect from NO are observed across several other host–symbiont and host–pathogen interactions, where NO serves as an important signaling and effector molecule (29, 30). For example, *Aliivibrio fischeri*, the symbiont of the Hawaiian bobtail squid *Euprymna scolopes* (reviewed in ref. 76) is exposed to host-derived NO (34), which contributes to ensuring host-symbiont specificity (33, 34). NO resistance in *A. fischeri* is mediated by upregulation of flavohemoprotein (*hmp*) (77), which is also up-regulated in *S. philanthi* upon NO exposure, and detoxifies NO by converting it to nitrate (58). Flavohemoprotein is also essential for NO resistance of nitrogen-fixing symbionts in the legume-rhizobium symbiosis, where NO constitutes an important signaling molecule (78, 79).

In the intracellular pathogen *Mycobacterium tuberculosis,* the causative organism of tuberculosis (80), NO exposure in vitro triggers responses to ROS and reactive radicals in general. This includes the upregulation of thioredoxin- and thioredoxin reductase genes (*thiX*, *trxB1*, *trxC*; *trxB2*) (69), while genes implicated in the protection from nitrosative and oxidative stress show high constitutive expression, particularly superoxide dismutase (*sodA*, *sodC*) and alkyl hydroperoxide reductase (*ahpD*, *ahpE*, *ahpC*) (69). Similarly, we observed high constitutive expression of genes with superoxide dismutase function (*sodF*, *sodN1*, *sodN2*) in *S. philanthi* in vitro, whereas genes with alkyl peroxidase function (e.g., *ahpC*) were down-regulated. Furthermore, genes for iron–sulfur and cysteine biosynthesis were up-regulated in *M. tuberculosis* in an effort to replace degraded iron–sulfur clusters (69). We also observed an upregulation of these genes in *S. philanthi* in vitro.

In the natural environment of beewolf brood cells, *S. philanthi* does not appear to suffer high mortality upon exposure to NO released by the beewolf egg, as previous studies indicate that a large proportion of the symbiont population in the brood cell is later transferred to the cocoon (81). Concordantly, within beewolf brood cells, *S. philanthi* did not exhibit the profound gene expression changes upon NO exposure that we observed in vitro. Nevertheless, multiple chaperones were up-regulated, and several catalases, oxidoreductases, bacterioferritin, and a cold shock protein exhibited high constitutive expression levels. Furthermore, the AGS proteome contained many symbiont-encoded proteins that are putatively involved in protection from nitrosative and oxidative stress, including flavohemoprotein, ferredoxin, thioredoxin and thioredoxin reductase, various catalases, and molecular chaperones, as well as superoxide dismutase and enzymes connected to iron uptake and homeostasis. Thus, the biosynthesis of proteins that scavenge NO or alleviate its detrimental effects is likely important for enhancing symbiont survival in the natural setting. However, the high mortality upon exposure to ecologically relevant NO concentrations in vitro indicates that host factors are necessary to protect the symbiont from the host-emitted NO in the brood cell.

### Host-Mediated Protection from NO.

To assess whether *P. triangulum* may protect *S. philanthi* from NO-inflicted cellular damage, we first screened the AGS proteome for proteins with putative protective functions against NO. Indeed, our proteome analysis revealed the presence of a host-derived superoxide dismutase, a heat shock protein of insect origin, as well as a glutathione synthase, a glutathione S-transferase, and a glutathione peroxidase. Glutathione is a low-molecular-weight thiol with antitoxic, antioxidant and modulator properties, and glutathione-mediated reactions protect bacteria from RNS and ROS (57, 82, 83). While glutathione synthase converts the precursor glutamyl-cysteine to glutathione, glutathione S-transferase catalyzes glutathione conjugation to endogenous metabolites or xenobiotic substances for detoxification purposes, and glutathione peroxidase performs the glutathione-dependent reduction of hydroperoxide (83). These findings suggest that *P. triangulum* secretes proteins that help to protect its symbiont from the host’s own NO defense.

Considering the high mortality of *S. philanthi* upon NO exposure in vitro, we hypothesized that the AGS might additionally shield *S. philanthi* from NO by acting as a diffusion barrier. In line with this hypothesis, our in vivo assays revealed that the application of the AGS to an iodide-starch indicator filter paper in a beewolf brood cell protected the covered region from oxidation. Our findings provide evidence for *P. triangulum* adaptations to protect *S. philanthi* from the host’s NO defense mechanism through the physicochemical prevention of NO diffusion into the aqueous milieu of the symbiont cells in the AGS. The AGS has previously been described to serve a dual function: First, it provides the larva with directional information for proper cocoon spinning and later emergence (84). Second, it mediates transmission of the symbiont to the larva to incorporate into the cocoon for protection against infection (46). Our study indicates that the latter function is supported by an additional trait of the AGS, i.e. the physicochemical protection of the symbiont from host-emitted NO.

The AGS contains high amounts of long-chain saturated and unsaturated hydrocarbons (53). Thus, we hypothesized that these compounds might be responsible for preventing NO diffusion. Concordantly, beewolf CHC hexane extracts with a composition comparable to the AGS, but without the background of symbiont- and host-encoded proteins of the AGS, prevented diffusion of NO into a liquid indicator solution in vitro. Furthermore, synthetic (Z)-9-tricosene, a constituent of the AGS, rescued *S. philanthi* survival upon exposure to otherwise lethal NO concentrations in vitro, albeit in higher concentrations than the approximated HC concentrations in the AGS, due to the need to apply the HC without a harmful solvent.

While the AGS hydrocarbons have been hypothesized to serve as an olfactory cue to help the larva localize the AGS, and/or as a nutritional resource for *S. philanthi* (53), our results indicate that they play an important role in protecting the symbionts from the detrimental effects of the host-produced NO. Considering that the hydrocarbon composition of NO-exposed AGS (53) closely resembles that of the postpharyngeal gland (PPG), the hemolymph, and the cuticle of *P. triangulum* (53, 74, 75, 85), the protective effect is likely due to the physical properties of forming a hydrophobic barrier impenetrable to the water-soluble NO, rather than a chemical mechanism of radical scavenging. Consistent with this hypothesis, SEM images of the AGS revealed that the secretion entirely covers the symbiont cells. CHCs have long been known to protect insects from environmental stress, including desiccation and pathogen attack (reviewed in refs. 86 and 87). Secondarily, CHCs have often evolved to serve as signals in chemical intraspecific and interspecific communication, particularly in social insects (reviewed in refs. 86 and 87), or as a physicochemical mechanism to reduce water condensation and thereby delay fungal growth (43). Our description of hydrocarbons protecting an insect symbiont from the host’s own defenses adds another facet to the diverse array of functions fulfilled by insect CHCs.

Most extracellular symbionts in insects experience periods of exposure to the environment during transmission across host generations (16). Especially considering the commonly observed patterns of genome erosion and metabolic streamlining in vertically transmitted symbionts (88–90), host adaptations for symbiont protection from abiotic and biotic stressors are likely common, but remain poorly studied to date. Recently, however, a host adaptation to protect its symbiont from the harsh conditions during the vulnerable period outside of the host environment has been described in plataspid stinkbugs (91). These bugs are obligately associated with *Ishikawaella*, a gut symbiont with a severely reduced genome (92, 93). During its vertical transmission via specialized symbiont capsules, *Ishikawaella* is subjected to intense sunlight for seven to 10 days (94). Within the capsule, *Ishikawaella* cells are embedded in a special intestinal protein provided by the females, which ensures survival during transmission and successful establishment in the offspring, but the mechanism of protection remains elusive (91). The widespread occurrence of extracellular transmission routes on the surface or in the vicinity of eggs across many Hemiptera, Coleoptera, some Hymenoptera and Diptera, as well as other insect orders (16) highlights the need for protective adaptations from the symbiont or host side to buffer environmental challenges and sustain a long-term stable symbiotic association.

## Conclusion

During transmission, the beewolf’s AGS containing the defensive symbiont *S. philanthi* is exposed to toxic nitric oxide (NO) that the host produces to reduce the growth of harmful fungi in the brood cell. In vitro assays show that the symbiont mounts a global stress response upon NO exposure, but is unable to survive concentrations of the gaseous radical that mimic those within the beewolf brood cell. Instead, our results reveal that the beewolf protects *S. philanthi* from oxidative and nitrosative damage by producing protective enzymes and embalming the symbiont in a secretion containing long-chain hydrocarbons. These hydrocarbons block diffusion of the toxic NO, adding yet another dimension to the diverse array of functions fulfilled by insect hydrocarbons. In light of our experiments, the beewolf–*Streptomyces* symbiosis presents an interesting example of a host adaptation to protect its symbiont from the host’s own immune defense, thereby ensuring reliable vertical transmission and long-term stability of the symbiosis.

## Material & Methods

### *Streptomyces* Cultivation.

*S. philanthi* 23Af2 was cultivated in vitro following a previously published protocol (95). An inoculum of *S. philanthi* from a glycerol stock was grown without shaking in liquid Grace medium (ThermoScientific, Germany). Grace medium base was dissolved in 1 L distilled water and supplemented with 50 µL phenol red, tryptose-phosphate broth (final concentration of 0.2 g/L tryptose, 0.02 g/L dextrose, 0.05 g/L NaCl, 0.025 g/L disodium phosphate; all from Carl-Roth, Germany) and the pH was adjusted to pH 6.5 with 1 M NaOH (Carl-Roth, Germany). *Streptomyces coelicolor* was cultured in YEME broth (3 g/L yeast extract, 3 g/L malt extract, 5 g/L peptone; all from Carl-Roth, Germany). To obtain spores, an inoculum was cultivated on YEME agar (same as YEME broth with the addition of 15 g/L agar; Carl-Roth, Germany) for 14 d at 25 °C. After sporulation occurred, spores were harvested in 0.05% Triton X-100 and stored in glycerol stocks (0.025% Triton X-100, 50% Glycerol; all from Carl-Roth, Germany) at −80 °C.

### Symbiont Survival upon NO Exposure In Vitro.

We used 7-d-old *S. philanthi* cultures grown at 25 °C as described above, with the addition of chicken egg lysozyme (7.5 µg/mL) to the cultivation medium to obtain short filamentous single cells. Similar growth stages occur in vivo in beewolf female antennae before secretion and NO exposure in natural brood cells (46). For *S. coelicolor* experiments, spores from a glycerol stock (see above) were washed twice with YEME broth.

Three technical replicates of each species were inoculated on solidified Grace agar (same composition as liquid grace medium described above, with addition of 10 g/L agar; Carl-Roth, Germany) and YEME agar, respectively. Two petri dishes were prepared for each species. The petri dishes were filled with 30 mL agar and had 60 mL air volume left. Both species were incubated at 25 °C for one more day.

In the brood cell, initial concentrations of NO are low. However, the egg emits a concentrated NO burst within 24 h after oviposition at temperatures above 24 °C, with the majority of NO being released within a 2 h period (39). In order to mimic the conditions in the beewolf brood cell, we assessed at which NO concentration we achieved antifungal effects that were equivalent to those obtained with an exposure to a beewolf egg (39). We found that replacing half of the remaining headspace of a petri dish with 1% NO in N_2_ 10 times over the course of 1 h reproduced the antifungal effect of a beewolf egg on inoculated fungal spores in vitro. We used this activity-guided approach henceforward for bacterial exposure to NO in vitro. While this procedure theoretically results in a concentration of 5,000 to 9,990 ppm in the petri dish, losses due to the reaction of NO with the plastic and diffusion into the agar medium likely reduce the concentrations to levels that have been recorded for beewolf brood cells in vivo (1,690 ± 680 ppm, maximum 4,448 ppm) (39).

Colonies were exposed to either 1% NO in N_2_ (purity NO: 2.0; purity N_2_: 5.0; 2% error tolerance; Westfalen AG, Germany) or pure N_2_ (purity 5.0, Westfalen AG, Germany). Prior to gas exposure, petri dishes were sealed with parafilm (Bemis, USA), pierced in the center with a hot needle to allow for gas application and resealed with adhesive tape (Tesa, Germany). The 60 mL air volume of the petri dishes was displaced 10 times with 30 mL fresh NO/N_2_ or N_2_ every 6 min. Gas-tight glass syringes with Teflon stoppers (Hamilton, USA) were used to apply NO/N_2_ or N_2_.

The three replicates of one dish were assessed for bacterial survival consecutively at 0 h, 6 h, and 24 h after the exposure with random assignment of the single colonies to the three time points. We used the LIVE/DEAD™ BacLight™ Bacterial Viability Kit (ThermoScientific, Germany) that consists of propidium iodide to stain dead or dying bacteria with compromised cell membranes, and SYTO9 to stain cells with intact cell membranes. 5 µL of a solution of 1% of each dye in distilled water was added on top of a colony 15 min before imaging. Imaging was conducted with a Thunder Imaging System (Leica, Germany) using a 20×/0.80 HC PL APO objective and a monochrome CMOS camera (DFC9000GT-VSC13735). The propidium iodide signal was acquired with the 475-nm LED at 50% power, the DFT51010 filter cube, and a 535-nm fast emission filter. The SYTO9 signal was acquired with the 555-nm LED at 50% power, the DFT51010 filter cube and a 590-nm fast emission filter. As background fluorescence varied between species, treatments and time points, we optimized exposure and threshold settings individually to obtain visibly stained cells in both channels. Further, we applied the instant computational clearing algorithm with standard settings to reduce background fluorescence.

### Symbiont gene Expression upon NO Exposure In Vitro.

To assess the symbiont’s transcriptomic response to NO exposure in vitro, we generated six technical replicates per *Streptomyces* species and gas treatment (NO vs. N_2_). For each *S. philanthi* replicate, an inoculum from 7-d-old liquid cultures growing at 25 °C was transferred to fresh solid medium 1 wk before NO exposure. Cells were collected from liquid media by mild centrifugation (1,000 rpm for 4 min) and omission of the supernatant. The concentrated cell suspension was transferred in 20-µL droplets to individual wells of 24-well plates. For each *S. coelicolor* replicate, an inoculum of spores was washed twice with YEME broth and transferred onto solid medium. The wells of the multi-well plate had a total volume of 3 mL, and contained 1 mL of solid medium and 2 mL headspace. We used Grace agar (see above) for *S. philanthi* and YEME agar (see above) for *S. coelicolor*. *S. philanthi* cultures were incubated for 7 d at 25 °C, *S. coelicolor* for one day, due to its much faster growth. Prior to gas exposure, plates were sealed with adhesive plastic foil (VWR, Germany) and resealed with adhesive tape (Tesa, Germany) after piercing the foil with syringes during gas application. Colonies were exposed to either 1% NO in N_2_ (purity NO: 2.0; purity N_2_: 5.0; 2% error tolerance; Westfalen AG, Germany) or pure N_2_ (purity 5.0, Westfalen AG, Germany). The 2 mL air in the headspace of the 24-well plate wells was displaced 10 times with 1 mL fresh NO/N_2_ or N_2_ every 6 min. We used gas-tight glass syringes as described above.

Bacterial biomass was scraped off the agar plates using a sterilized spatula and shock-frozen in liquid nitrogen. Three of the technical replicates per species and treatment were harvested 2 h after exposure, the remaining three 6 h after exposure. RNA was extracted after all treatments were collected by lysing cells in 100 µL TE25S buffer (25 mM Tris-HCl [pH 8], 25 mM EDTA [pH 8], 0.3 M sucrose) containing 2 mg/mL lysozyme for 60 min at 37 °C. Lysates were further processed using the innuPREP DNA/RNA Mini Kit (Analytik Jena, Germany) following the manufacturer’s protocol. RNA was eluted with 30 µL RNase-free water and quantified using a NanoDrop1000 (Peqlab, Germany). 1.2 to 1.5 µg of total RNA was used for a further DNase digestion with 5U DNase (Qiagen, Germany) for 15 min at 37 °C, followed by a 10-min DNase inactivation step at 75 °C.

Library preparation and sequencing was performed at the Max Planck-Genome-Centre Cologne, Germany (https://mpgc.mpipz.mpg.de/home/). All replicates were subjected to rRNA depletion with the Ribo-zero magnetic gold kit for bacteria (Illumina), and then a directional RNAseq library was prepared with the NEBNext Ultra™ Directional RNA Library Prep Kit for Illumina (NEB) followed by sequencing as 100-bp single-end libraries at a sequencing depth of 4 million reads per sample on a HiSeq2500 device (Illumina, USA). Sequences were quality-controlled and trimmed using FastQC (96) and Trimmomatic (97). The retained reads were mapped to either the *S. coelicolor* or *S. philanthi* genome (50) using Bowtie2 (v.2.3.2) and StringTie (v.1.3.3b) implemented in KBase (98) using default settings. rRNA sequences were removed from the dataset before differential gene expression was analyzed using DESeq2 (v.1.22.2) (99) in RStudio (v1.1.453 with R v3.5.0). During the analysis, transcripts with less than 30 counts were discarded. Genes with significant difference in gene expression between NO and N_2_ treatments (FDR-adjusted *P*-values following Benjamini–Hochberg of Wald test < 0.05) and at least twofold expression difference were considered differentially expressed. For subsequent visualization of the data in ggplot2, LFC shrinkage was applied using the “apeglm” shrinkage estimator. Homologous genes were identified by reciprocal mapping of relevant genes on the genomes of *S. philanthi* and *S. coelicolor* in Geneious Prime (V2021.1.1).

## Beewolf Cultivation for In Vivo Bioassays.

Females of the European beewolf *P. triangulum* were collected in Berlin (Germany) for the proteome experiments and Mainz (Germany) for the in situ reaction of *S. philanthi* to NO released by the beewolf egg. Females were kept in observation cages (84) at 24 to 27 °C with a ~16/8 h day/night cycle, and provided ad libitum with honey and workers of the European honeybee *Apis mellifera*. The nesting compartment was covered with glass plates to allow for observation of the underground nesting behavior (*SI Appendix*, Fig. S12). A plastic foil was introduced below the glass cover to enable the sampling of the AGS from the brood cell ceiling by cutting out the respective piece of foil containing the AGS, as the AGS is visible with to naked eye as up to ten whitish spots on the plastic foil.

## Proteome of Symbiont-Containing Brood Cell Secretion (AGS).

AGS samples from fifty brood cells were collected at random time points within the approximately 10 d between females finishing a brood cell and larvae starting to spin their cocoon, and frozen at −80 °C until analysis. After thawing, samples were combined and suspended in 50 µL PBS, vortexed vigorously, and centrifuged for 10 min at 6,000 g to separate soluble proteins from cellular debris. 200 μL of rehydration buffer was added to sample supernatant (7 M urea, 2 M thiourea, 2% Chaps, 0.75% IPG buffer 3–11, bromophenol blue). Samples were immediately adsorbed onto 11 cm immobilized pH 3–11 gradient (IPG) strips (GE Healthcare Bio-Sciences) and rehydrated overnight. Isoelectric focusing was performed on an Ettan IPGphor II (Amersham plc) unit by using the following program: 500 V for 1 h, 500 to 1,000 V for 1 h, 1,000 to 6,000 V for 2 h, 6,000 V for 40 min. After focusing, the IPG strips were equilibrated for 15 min in 10 mL of equilibration buffer containing 6 M urea, 30% [v/v] glycerol, 2% [w/v] SDS, 75 mM Tris–HCl, 0.002% [w/v] bromophenol blue and 1% (w/v) DTT. Then, the strips were incubated for 15 min in the same buffer containing 2.5% (w/v) iodoacetamide instead of DTT. For the separation of proteins in the second dimension a 12.5% Criterion Tris-HCl Gel (Biorad) was used. The proteins were separated for 3 h at 100 V and stained with Roti Blue (Carl Roth) and visualized by a densitometer (Biorad GS-800).

The protein sample was diluted in Roti-Load buffer (Carl Roth) and loaded on a 12.5% Criterion Tris-HCl precast 1D gel (Bio-Rad) according to the manufacturer's instructions. Proteins were allowed to migrate for 1 h at a constant voltage of 200 V. After migration, the gel was stained overnight with Roti Blue (Carl Roth) staining solution, rinsed twice with deionized water, destained in 25% methanol, and scanned using a densitometer (Biorad GS-800). Protein bands/spots were cut from the gel matrix and tryptic digestion was carried out as described (100). For LC-MS analysis, the extracted tryptic peptides were reconstituted in 10 µL aqueous 1 % formic acid. Depending on staining intensity, 1 to 5 µL of sample was injected into the LC-MS/MS system. The samples were acquired on a nanoAcquity UPLC system online connected to a Q-ToF Synapt HDMS mass spectrometer (Waters). The peptides were concentrated on a Symmetry C18 trap-column (20 × 0.18 mm, 5 µm particle size) using a mobile phase of 0.1% aqueous formic acid at a flow rate of 15 µL/min and separated on a nanoAcquity C18 column (200 mm × 75 µm ID, C18 BEH 130 material, 1.7 µm particle size) by in-line gradient elution at a flow rate of 0.350 µL/min using the following gradient: 1 to 15% B over 5 min, 15 to 40% B over 25 min, 40 to 55% B over 10 min, 55 to 80% B over 10 min, 80 to 95% B over 1 min, isocratic at 95% B for 1 min, and a return to 1% B (Buffers: A, 0.1 % formic acid in water; B, 100 % acetonitrile in 0.1 % formic acid).

Data were acquired using data-dependent acquisition (DDA) and data-independent acquisition (DIA, referred to as enhanced MSE). The acquisition cycle for DDA analysis consisted of a survey scan covering the range of *m/z* 400-1800 Da followed by MS/MS fragmentation of the five most intense precursor ions collected at 1.5-s intervals in the range of 50 to 2,000 *m/z*. Dynamic exclusion was applied to minimize multiple fragmentations for the same precursor ions. For LC-MSE analyses, full-scan LC-MS data were collected using alternating modes of acquisition: low-energy (MS) and elevated-energy (MSE) modes at 1.5 s in the range *m/z* of 50 to 1,900. The collision energy of low-energy MS mode and high-energy mode (MSE) were set to 4 eV and 20 to 40 eV energy ramping, respectively. A reference compound, human Glu-Fibrinopeptide B [650 fmol/mL in 0.1 % formic acid/ACN (v/v, 1:1)], was infused through a reference sprayer at 30-s intervals for external calibration. The data acquisition was controlled by MassLynx v4.1 software (Waters).

DDA raw data were processed and searched against a subdatabase containing common contaminants (human keratins and trypsin) using ProteinLynx Global Server (PLGS) version 2.5.2 (Waters). The following search parameters were applied: fixed precursor ion mass tolerance of 10 ppm for survey peptide, fragment ion mass tolerance of 0.02 Da, estimated calibration error of 0.002 Da, 1 missed cleavage, fixed carbamidomethylation of cysteines, and possible oxidation of methionine. Spectra remaining unmatched by database searching were interpreted de novo to yield peptide sequences and subjected to homology-based searching using MS BLAST program (101) installed on a local server. MS BLAST searches were performed against beewolf and *Streptomyces* subdatabases both obtained from in silico translation of beewolf and *Streptomyces* transcriptome data and against insect and bacteria databases downloaded from https://www.ncbi.nlm.nih.gov/ on June 02, 2017. In parallel, pkl-files of MS/MS spectra were generated and searched against beewolf and *Streptomyces* subdatabases combined with NCBInr database (https://www.ncbi.nlm.nih.gov/, downloaded on January 19, 2017, containing 112,583,993 sequences) using MASCOT software version 2.6.2.

The acquired continuum LC-MSE data were processed using ProteinLynx Global Server (PLGS) version 2.5.2 (Waters). The thresholds for low-/high-energy scan ions and peptide intensity were set at 150, 30, and 750 counts, respectively. The processed data were searched against beewolf and *Streptomyces* protein subdatabases combined with Swissprot database downloaded from http://www.uniprot.org/. The database was searched at a false discovery rate (FDR) of 4%. The minimum number of product ion matches per peptide was set to 3, and the minimum number of product ion matches was set to 7. The minimum number of peptide matches and the maximum number of missed tryptic cleavage sites were both set to 1. Searches were restricted to tryptic peptides with a fixed carbamidomethyl modification for Cys residues.

## Symbiont Gene Expression upon NO Exposure in Beewolf Brood Cells.

NO emission by the beewolf egg starts approximately 16 to 24 h after oviposition at our beewolf rearing temperature of 24 °C to 27 °C (39). To examine the effects of NO exposure on symbiont gene expression in the AGS, brood cells with one or two bees were manipulated within 1 h after oviposition. Each brood cell was checked for premature NO emission via the characteristic sharp, sweet NO smell. To obtain an NO-unexposed sample, the bee holding the beewolf egg was removed from the brood cell and discarded. To obtain NO-exposed samples, the bee carrying the egg was removed and then reintroduced into the respective brood cell after a 1-min removal. After 24 h, the piece of plastic foil bearing the AGS of both treatments was cut out and transferred to a 1.5-mL reaction tube. Given the 16 to 24 h that it takes until NO emission by the beewolf egg and the manipulation of the brood cell within 1 h after oviposition, sampling 24 h after manipulation corresponds to an average of 4 to 5 h after NO exposure. Samples were frozen in liquid nitrogen and stored at −80 °C.

RNA was extracted from five NO-exposed and five NO-unexposed AGS samples using the Epicentre MasterPureTM DNA and RNA kit (Epicenter Technologies, Madison, USA). Extraction was carried out according to the manufacturer’s instruction with the following modifications: Pieces of plastic foil with the AGS were transferred to 100 μL TE25S buffer (25 mM Tris [pH 8], 25 mM EDTA [pH 8], 0.3 M sucrose) with 50 µL lysozyme (50 mg/mL) and incubated at 37 °C for 1 h at 750 rpm. Afterward, the plastic foil was removed from the samples, and the crude extracts were processed following the manufacturer’s instructions. The extracted nucleic acids were resuspended in 20 μL TE buffer. DNA was digested using PerfeCTa DNase I (Quantabio, Beverly/MA, USA) according to the manufacturer’s instructions. RNA samples were sequenced at the Max Planck-Genome-Centre Cologne, Germany (https://mpgc.mpipz.mpg.de/home/). All replicates were subjected to rRNA depletion. Due to low-input amounts of RNA, rRNA was depleted on the cDNA level using oligonucleotides complimentary to *S. philanthi* rRNA (Tecan Genomics, Switzerland). All replicates were sequenced individually as 150-bp paired-end libraries with a sequencing depth of 2 million reads per sample on a HiSeq3000 system (Illumina, USA). Sequences were processed and analyzed as described above for the in vitro dataset, with the exception of only omitting transcripts with less than five counts.

## Effect of NO Exposure on Symbiont Titer and Antibiotic Production on the Cocoon.

To examine whether NO exposure in the brood cell impacts subsequent symbiont growth and antibiotic production on the cocoon, we sampled cocoons with previously NO-exposed and NO-unexposed symbionts, respectively. After oviposition, the piece of plastic foil with AGS was removed and transferred to an artificial brood cell in a control cage. In this set-up, we removed the plastic foil to enable brood cell sanitation by NO emission, so that the larval provisions would not be overgrown by fungi over the course of the experiment. To obtain cocoons with NO-exposed symbionts, the AGS was removed and then reintroduced into the respective brood cells after ~1 min. To obtain cocoons with NO-unexposed symbionts, the AGS was transferred to an artificial brood cell and reintroduced into its original brood cell after 24 h, i.e. after the beewolf-produced NO had already diffused into the surrounding sand. The resulting cocoons were harvested 7 d after cocoon spinning, since the amount of antibiotics reaches a peak around this time (55). We measured length and width of a random subset of the cocoons, dissected out the pupae and flash-froze the cocoons in liquid nitrogen before storage at −80 °C. We used a paired design, sampling one cocoon with NO-exposed symbionts and one cocoon with NO-unexposed symbionts per female, to account for genetic differences between individuals. We collected nine sample pairs in total, and measured the length and width for a subset of five random sample pairs. We approximated the cocoon surface by the product of length and width to calculate the amount of antibiotics in relation to the cocoon area.

The amounts of the three major compounds (streptochlorin, piericidin A1 and B1) (47) of the symbiont-produced antibiotic cocktail were quantified as previously described (55). The cocoons were thawed and then individually submerged in HPLC grade methanol (Carl-Roth, Germany) with 2 µg of internal standard (octadecan-1-ol in hexane, Sigma-Aldrich, Germany) for 60 min at 350 rpm shaking to extract antibiotic compounds. After removing the cocoons, the solvent was completely evaporated under a nitrogen stream, and residual compounds were redissolved in 50 µL methanol. Aliquots of 1 μL of each sample were injected into a Varian 450GC gas chromatograph coupled to a Varian 240MS mass spectrometer (Agilent Technologies, Böblingen, Germany) using a split/splitless injector operated at 250 °C in splitless mode. The GC was equipped with a DB5-MS capillary column (30 m × 0.25 mm diameter, film thickness: 0.25 μm, Agilent Technologies) and programmed from 150 to 300 °C at 5 °C/min with a 1-min initial and a 10-min final isothermal hold. Helium was used as carrier gas, with a constant flow rate of 1 mL/min. Mass spectra were recorded using electron ionization (EI-MS) in the external configuration of the ion trap held at 90 °C with a mass range of 45 to 500 *m/z*. Data acquisition and quantifications were achieved with the MS Workstation Version 6.9.3 Software (Agilent Technologies). A dilution series (1 to 500 ppm) of commercially available piericidin A1 was used as an external calibration standard for both piericidin derivatives, assuming similar ionization efficiencies based on the high structural similarity. For quantification of streptochlorin, we used a dilution series of a synthesized streptochlorin standard (55). The peaks were identified by comparison of their mass spectra with the standard spectra and with published reference spectra, and peak areas were manually integrated using the MS Workstation Software.

After antibiotic extraction, the same cocoons were used for DNA extraction (55), using the Epicentre MasterPureTM DNA extraction kit (Epicenter Technologies, Madison, USA). Extraction was carried out according to the manufacturer’s instructions with the following additional steps: To better lyse the gram-positive symbiont cells, cocoons were crushed and transferred to 100 μL Tissue and Cell Lysis solution with 8 μL lysozyme (100 mg/mL). Samples were incubated at 37 °C for 1 h. After protein precipitation, an additional centrifugation step (14,000 rpm, 2 min) was added to optimize the transfer of the supernatant. Extracted DNA was resuspended in 50 μL low TE buffer.

Symbionts on the cocoons were quantified via quantitative PCR (qPCR). The 16S rDNA of *S. philanthi* was amplified using specific primers Strep_phil_fwd3mod (5′ TGGTTGGTGGTGGAAAGC 3′) and S16S_rev (5′ GTGTCTCAGTCCCAGTGTG 3′) (81), resulting in a 135-bp product. Amplification was performed on a Rotor Gene Q cycler (Qiagen, Hilden, Germany), using 5x HOT FIREPol® EvaGreen® qPCR Mix Plus (Solis BioDyne, Tartu, Estonia). Each sample of extracted DNA was diluted 1:100 prior to qPCR. One reaction comprised 6 μL H_2_O, 2 μL EVA Green Mix, 0.5 μL of each primer, and 1 μL of the diluted DNA template. Samples were incubated at 95 °C for 10 min, followed by 40 cycles of 15 s at 95 °C, 30 s at 60 °C, and 20 s at 72 °C. A melting curve was recorded by increasing the temperature from 60 °C to 95 °C over a time period of 20 min. The purified target amplicon of the 16S rDNA primers served as the template in a dilution series from 1.48 ng/μL to 1.48 pg/μL. The number of gene copies was then calculated (1 ng of the amplified product equals 6,758,851,515 copies). The resulting symbiont titers, as well as antibiotic amounts, and antibiotic amounts standardized by cocoon area were compared between treatments by paired *t* tests using R V5.

## Preparation of NO Indicator.

We mixed 1 mL of a 10% (w/v) potassium iodide (Carl-Roth, Germany) solution with 5 mL of a 2% (w/v) boiled starch (Carl-Roth, Germany) solution and 4 mL of ultrapure water (Merck, Germany). Filter papers were soaked in this mixture and dried at 60 °C overnight. Dried filter papers were stored in glass containers flooded with N_2_ and stored until use. We employed this indicator to qualitatively visualize the presence of NO via detection of oxidation: NO or its spontaneous reaction products oxidize iodide to iodine. Elemental iodine then integrates into the starch double helix which results in a dark coloration (102).

## Protective Effect of the AGS for the Symbionts in the Brood Cell.

To test for a role of the AGS in protecting the embedded symbionts from NO exposure, we transferred freshly secreted AGS onto a piece of NO indicator filter paper and reintroduced it into the corresponding brood cell. We monitored changes in its coloration after NO emission by the beewolf egg, which we documented using a digital camera (Canon DS126311, Canon, Japan) and an X-Rite ColorChecker classic 24-color chart to standardize coloration between pictures. We further confirmed the presence and localization of symbiont-bearing AGS on the NO indicator, making use of its autofluorescence, with either a M165FC (Leica, Germany) or an Axio Zoom.V16 (Zeiss, Germany) and GFP filter sets. Samples were imaged without further treatment using reflective light and fluorescence illumination.

Standardized images of the NO-exposed and control indicators were evaluated in Photoshop CD5 V12.064. To compare the change in coloration between the colored area, the area bearing the AGS, and a control indicator paper, all images were first converted to grayscale. For each of these areas, we identified the mean gray value, i.e. the mean darkness of the selected pixels, using the wand tool with the default setting, and normalized it by the mean gray value of the respective imaging standard. We analyzed the results in R (V4.15) using a one-way ANOVA.

## Protective Effects of Beewolf Hydrocarbons from NO In Vitro.

The AGS contains high amounts of saturated and unsaturated linear hydrocarbons (53). Previous studies revealed similar hydrocarbon compositions of the AGS, the postpharyngeal gland (PPG), the hemolymph, as well as the epicuticle of female *P. triangulum* (53, 74, 75, 85). In addition, the hydrocarbon profiles of females and males were found to be comparable (103). Given the easier access to male beewolves than to AGS samples or female beewolves, we used CHC extracts of adult male beewolves to test for the protective activity of the beewolf CHCs against NO. To avoid contamination with the male sex pheromone (103), we removed the head from twelve male beewolves and extracted CHCs by immersing the two bodies per extract in hexane for 10 min, producing six replicate extracts. Wasp bodies were removed, and hexane was evaporated under a gentle flow of nitrogen. Each CHC extract was resuspended in 10 μL hexane and used to cover 40 μL of an iodide-starch indicator solution (see above) in a qPCR tube (diameter: 3 mm; Biozym, Germany). A control was prepared with a microcentrifuge tube containing 40 μL of the indicator solution and 10 μL hexane on top; six controls were generated. For treatments and controls, the hexane was allowed to evaporate. The tubes were positioned in a 24-well plate, and the well plate was placed in a sealed exposure chamber. Half of the remaining headspace was replaced with 0.01% NO in N_2_ every 6 min over the course of 1 h. We chose this concentration since this procedure resulted in clear NO detection, i.e. a complete change in coloration of the indicator solution, in a preliminary experiment. The qPCR tubes were then removed from the exposure chamber, and the indicator solution was transferred to 500-μL microcentrifuge tubes. After centrifugation at 10,000 g for 25 s, the supernatant was transferred to a 384-well plate and the absorbance at 540 nm was measured in a VarioSkan Lux (Thermo Fisher Scientific, Germany). We analyzed the results in R (V4.15) using the Wilcoxon rank sum exact test.

## Protective Effect of (Z)-9-tricosene on Symbiont Survival upon NO Stress In Vitro.

*S. philanthi* 23Af2 was cultured in a 1:1 mixture of Sf-900 II SFM medium (Gibco, Thermo Fisher Scientific, Germany) and Grace’s insect medium (see above) at 30 °C in 24-well plates. To test whether HCs can protect symbiont cells in vitro, we transferred 210 μL of a growing culture of *S. philanthi* to a 1.5-mL reaction tube and gently yet thoroughly resuspended it. After division into ten aliquots of 20 μL each, the bacterial cells were pelleted by centrifugation, and the supernatant was reduced. Five control aliquots were plated on 500 μL of a mix of Grace’s insect medium, Sf900 medium and 3% agar (ratio 1:1:2; pH 8) in a 48-well plate. The remaining aliquots were mixed with 20 μL (Z)-9-tricosene and then transferred to the same well plate. After 1-h incubation at RT, the plate was positioned in a sealed exposure chamber. Half of the remaining headspace was replaced with 1% NO in N_2_ every 6 min over the course of 1 h. Afterward, (Z)-9-tricosene was removed with a pipette. The cultures were monitored for macroscopically visible growth for 8 d.

## Ultrastructure of *S. philanthi* In Vitro and in the AGS.

*S. philanthi* 23Af2 in vitro cultures from Grace’s insect medium were subjected to SEM as described previously (95). Similarly, AGS applied by beewolf females to plastic foils that had been introduced into beewolf brood cells (see above) were harvested from three brood cells and inspected by SEM as described previously (45).

## Supplementary Material

Appendix 01 (PDF)

Dataset S01 (XLSX)

## Data Availability

Transcriptome sequencing data have been deposited in the NCBI database under accession numbers BioProject PRJNA975590 (104), BioSamples SAMN35335670–SAMN35335672 (105–107), SRAs SRX20486080–SRX20486113 (108–141). Proteomics data are available from the Open Research Data Repository Edmond of the Max Planck Society (https://dx.doi.org/10.17617/3.5r) (70).
