# Supplementary material for: Host hydrocarbons protect symbiont transmission from a radical host defense
Source: Proc Natl Acad Sci U S A. 2023 Jul 24;120(31):e2302721120. doi: 10.1073/pnas.2302721120 (PMC10400980; doi:10.1073/pnas.2302721120)
Supplement: Supplementary file 1 — Appendix 01 (PDF) [file pnas.2302721120.sapp.pdf]

PNAS

Supporting Information for

Host hydrocarbons protect symbiont transmission from a radical host defense

Chantal Selina Ingham[1,§], Tobias Engl[1,2,§], Bernal Matarrita-Carranza[2], Paul Vogler[1], Bruno Huettel[3], Natalie Wielsch[4], Aleš Svatoš[4], Martin Kaltenpoth[1,2,*]

[1]Department of Evolutionary Ecology, Institute of Organismic and Molecular Evolution, Johannes Gutenberg-University Mainz, 55128 Mainz, Germany
[2]Department of Insect Symbiosis, Max-Planck-Institute for Chemical Ecology, 07745 Jena, Germany
[3]Max Planck Genome Centre Cologne, Max Planck Institute for Plant Breeding Research, 50829 Cologne, Germany
[4]Research Group Mass Spectrometry/Proteomics, Max-Planck-Institute for Chemical Ecology, 07745 Jena, Germany
[§]equal contributions

*corresponding author: Martin Kaltenpoth, kaltenpoth@mpg.ice.de

This PDF file includes:

Figures S1 to S12
Tables S1 to S7
SI References

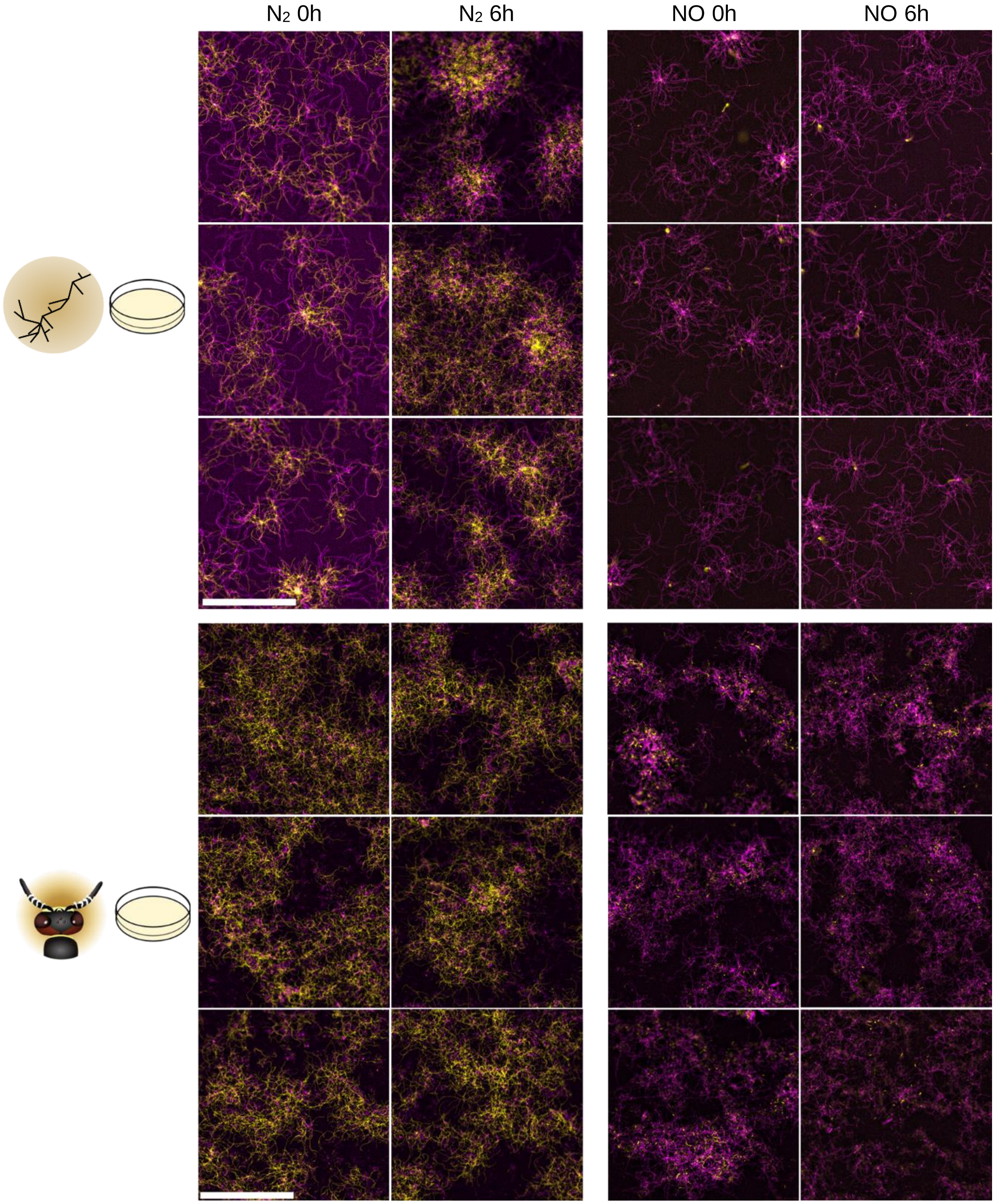

Fig. S1. Activity-guided survival assessment of free-living *S. coelicolor* (top, indicated by the symbol of filamentous bacteria) and symbiotic *S. philanthi* (bottom, indicated by the beewolf head) upon exposure to 1% NO diluted in N_2 or pure N_2 in vitro (indicated by the petri dish), using a fluorescent live-dead stain on a petri dish. Columns show fluorescent images of three technical replicates of *S. coelicolor* and *S. philanthi* 0h and 6h after N_2 or NO treatment, respectively. Yellow: SYTO9 stain of cells with intact cell membranes, i.e. living cells. Magenta: propidium iodide stain of cells with compromised cell membranes, i.e. dead cells. Scale bars: 200 µm.

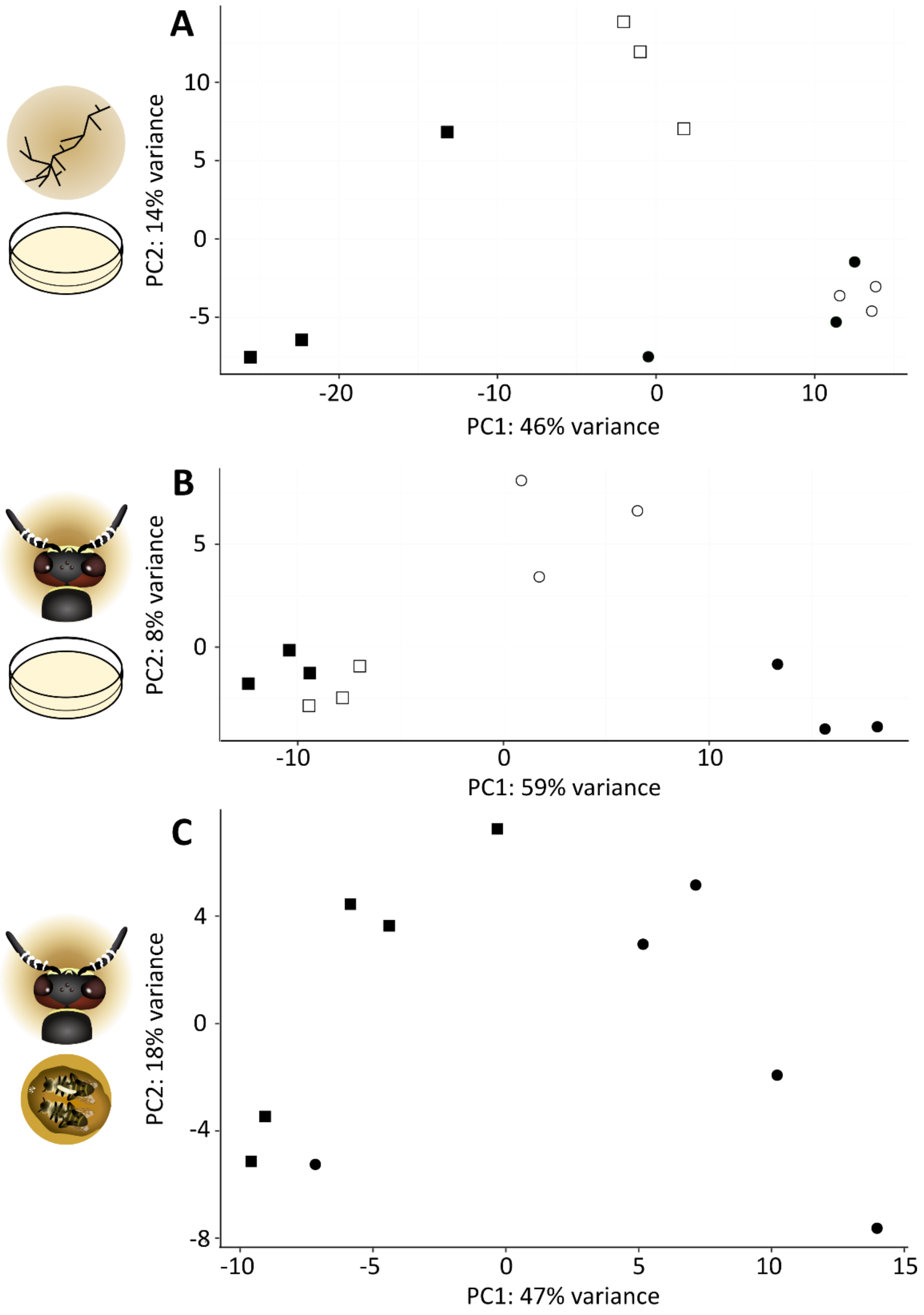

Fig. S2. PCA plots of transcriptome experiments of (A) free-living *S. coelicolor* (indicated by the symbol of filamentous bacteria), (B) symbiotic *S. philanthi* (indicated by the beewolf head) 2 h (non-filled symbols) and 6 h (filled symbols) after in vitro exposure (indicated by the petri dish) to pure N_2 (squares) or 1% NO diluted in N_2 (circles), and (C) symbiotic *S. philanthi* in antennal gland secretions incubated in beewolf brood cells (indicated by the beewolf brood cell) without (squares) or with an egg (circles). Expression values were rlog-transformed for PCA analysis.

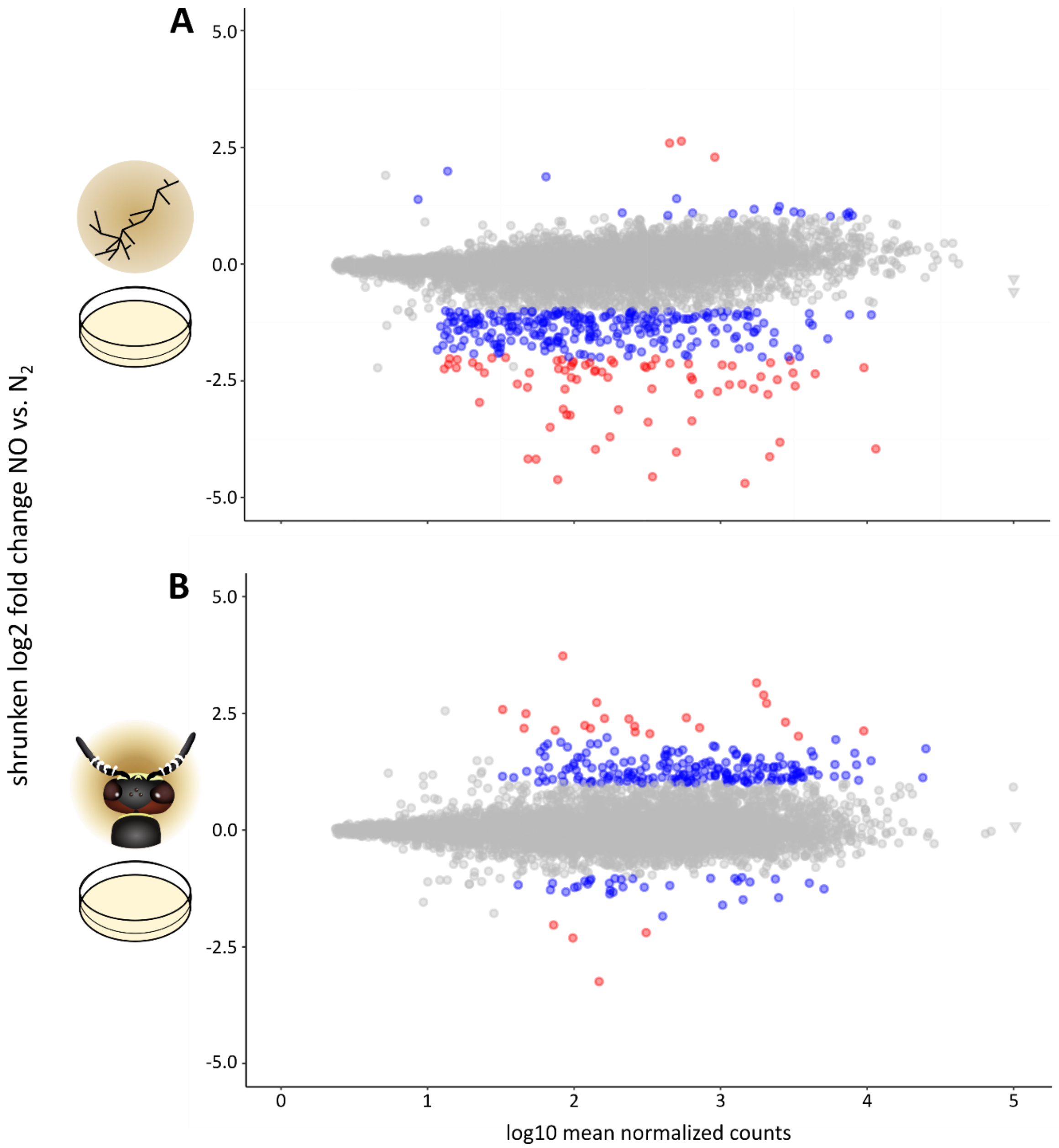

Fig. S3. Changes in gene expression in (A) free-living *S. coelicolor* (indicated by the symbol of filamentous bacteria) and (B) symbiotic *S. philanthi* (indicated by the beewolf head) 2 h after in vitro exposure (indicated by the petri dish) to NO in comparison to exposure to N_2. Significant gene expression differences are highlighted in color (adjusted $p<0.05$ and 2-4 fold differential expression in blue and more than 4-fold change in red). Log-transformed values of some extremely highly expressed genes were set to 5 to improve readability and are indicated with triangles instead of circles.

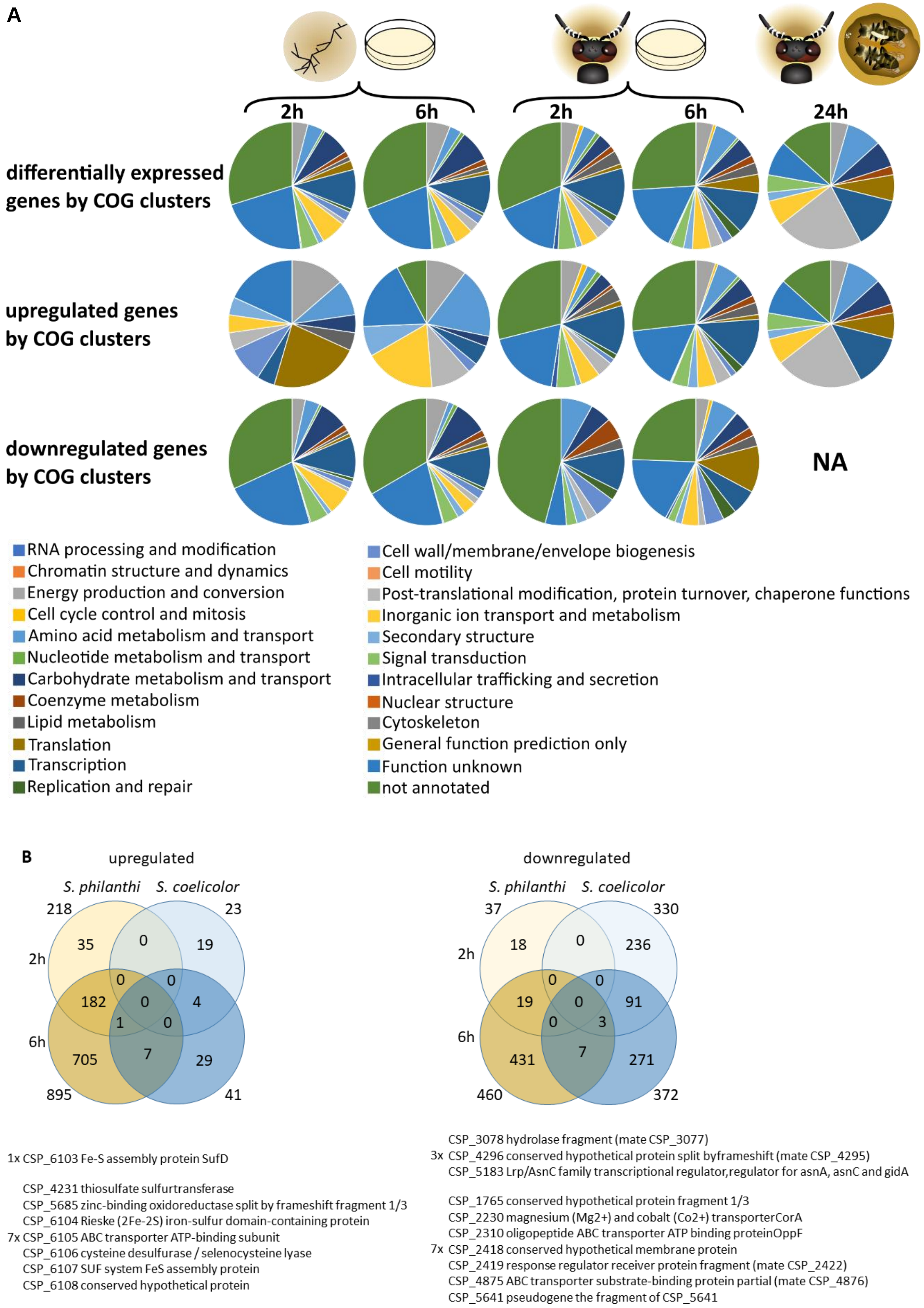
A
2h
6h
2h
6h
24h
differentially expressed genes by COG clusters
upregulated genes by COG clusters
downregulated genes by COG clusters
NA
RNA processing and modification
Chromatin structure and dynamics
Energy production and conversion
Cell cycle control and mitosis
Amino acid metabolism and transport
Nucleotide metabolism and transport
Carbohydrate metabolism and transport
Coenzyme metabolism
Lipid metabolism
Translation
Transcription
Replication and repair
Cell wall/membrane/envelope biogenesis
Cell motility
Post-translational modification, protein turnover, chaperone functions
Inorganic ion transport and metabolism
Secondary structure
Signal transduction
Intracellular trafficking and secretion
Nuclear structure
Cytoskeleton
General function prediction only
Function unknown
not annotated
B
upregulated
S. philanthi
S. coelicolor
218
23
2h
6h
35
0
19
0
0
182
0
4
1
0
705
7
29
895
41
downregulated
S. philanthi
S. coelicolor
37
330
2h
6h
18
0
236
0
0
19
0
91
0
3
431
7
271
460
372
1x CSP_6103 Fe-S assembly protein SufD
CSP_4231 thiosulfate sulfurtransferase
CSP_5685 zinc-binding oxidoreductase split by frameshift fragment 1/3
CSP_6104 Rieske (2Fe-2S) iron-sulfur domain-containing protein
7x CSP_6105 ABC transporter ATP-binding subunit
CSP_6106 cysteine desulfurase / selenocysteine lyase
CSP_6107 SUF system FeS assembly protein
CSP_6108 conserved hypothetical protein
CSP_3078 hydrolase fragment (mate CSP_3077)
3x CSP_4296 conserved hypothetical protein split byframeshift (mate CSP_4295)
CSP_5183 Lrp/AsnC family transcriptional regulator,regulator for asnA, asnC and gidA
CSP_1765 conserved hypothetical protein fragment 1/3
CSP_2230 magnesium (Mg2+) and cobalt (Co2+) transporterCorA
CSP_2310 oligopeptide ABC transporter ATP binding proteinOppF
7x CSP_2418 conserved hypothetical membrane protein
CSP_2419 response regulator receiver protein fragment (mate CSP_2422)
CSP_4875 ABC transporter substrate-binding protein partial (mate CSP_4876)
CSP_5641 pseudogene the fragment of CSP_5641

Fig. S4. (A) COG clusters of differentially expressed up-regulated and down-regulated genes of the gene expression analyses. The columns on the left and in the middle show COG clusters for free-living *S. coelicolor* (indicated by the symbol of filamentous bacteria) and symbiotic *S. philanthi* (indicated by the beewolf head) 2 h and 6 h after NO exposure (compared to N_2 exposure) in vitro (indicated by the petri dish). The columns on the right show COG clusters for symbiotic *S. philanthi* in antennal gland secretions incubated in beewolf brood cells for 24 h after oviposition (indicated by the beewolf brood cell). (B) Differentially expressed genes upon NO exposure versus N_2 exposure in *S. philanthi* and *S. coelicolor* 2 and 6 hours after exposure. The upper/lighter colored circles denote differentially expressed genes after 2h, the lower/darker colored circles after 6h, while orange colored circles denote *S. philanthi* and blue colored ones *S. coelicolor* differentially expressed genes. For the few genes that were differentially expresseds in both *S. coelicolor* and *S. philanthi*, the annotations based on the *S. philanthi* genome are listed below the Venn diagrams.

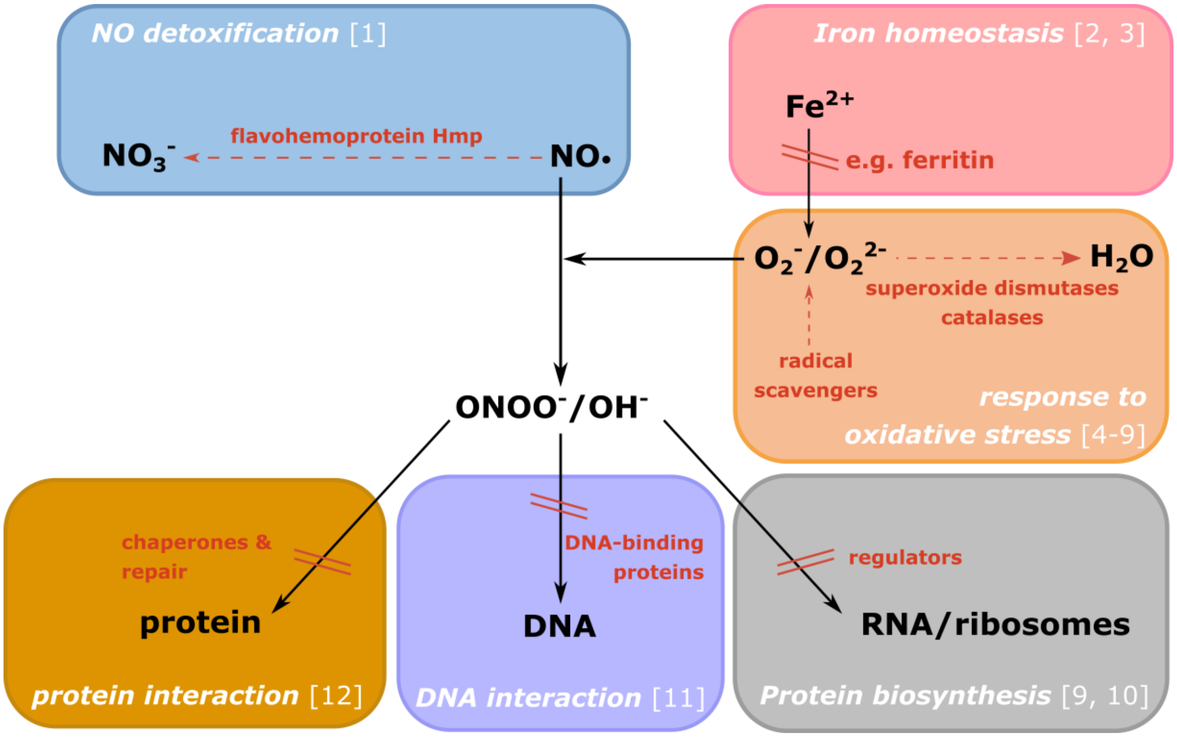

Fig. S5. Potential damage to different macromolecules inflicted by NO and NO-derived reactive nitrogen and oxygen species (black arrows), and cellular detoxification mechanisms (red). In addition to direct detoxification by flavohemoprotein, the detrimental effects of NO can be indirectly mitigated: Chaperones, DNA-binding proteins and other regulators attenuate damage to proteins and nucleic acids inflicted by NO-derived reactive nitrogen and oxygen species. To prevent the formation of these highly reactive species, ferrous iron and super- and peroxide can be intercepted. NO_3^- = nitrate, NO· = nitric oxide, Fe^{2+} = ferrous iron, O_2^- = superoxide, O_2^{2-} = peroxide, $ONOO^-$ = peroxynitrite, OH^- = hydroxide. References are given in square brackets. (1-12)

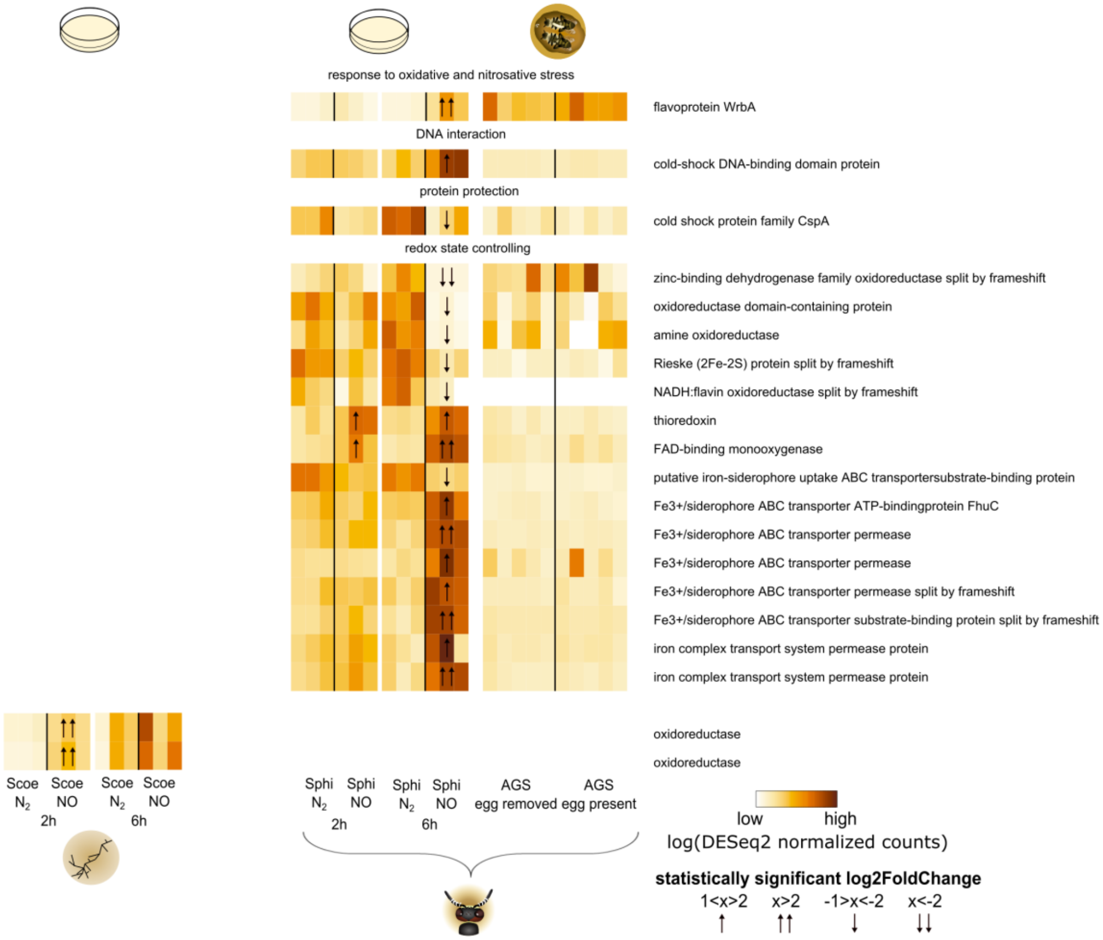

Fig. S6. Differentially expressed genes upon nitric oxide exposure in vitro that are unique to one of the bacterial strains. Scoe = free-living *S. coelicolor* (also indicated by the symbol of filamentous bacteria), Sphi = symbiotic *S. philanthi* (also indicated by the beewolf head), AGS = antennal gland secretion. The petri dish indicates gene expression in vitro, the beewolf brood cell symbolizes gene expression within the AGS.

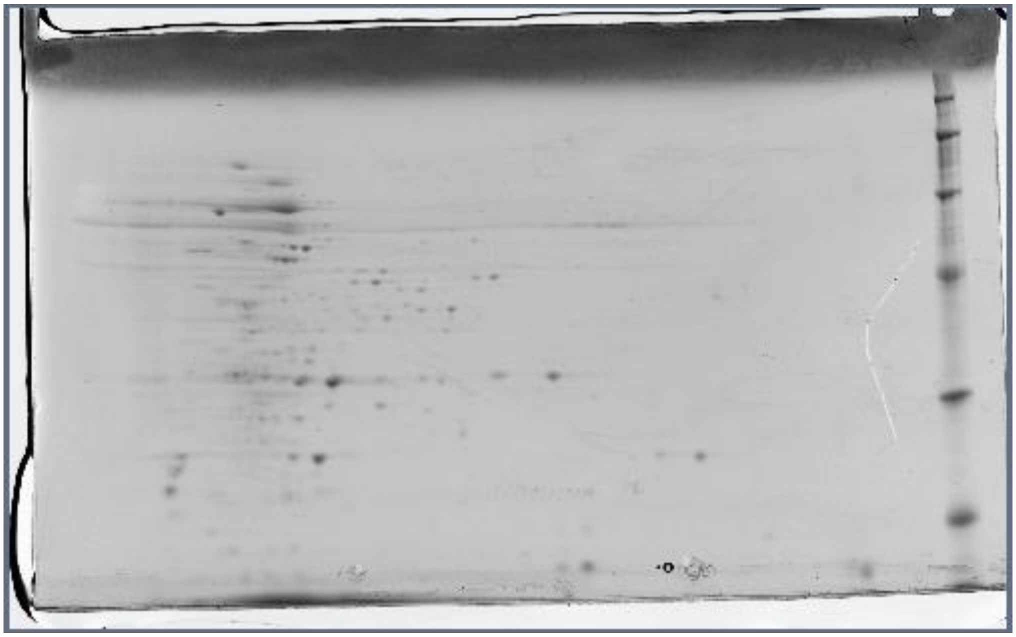

Fig. S7. 2D gel image of the soluble proteins extracted from a pooled sample of AGS from 70 beewolf brood cells. 90 spots were excised and used for LC-MS analysis.

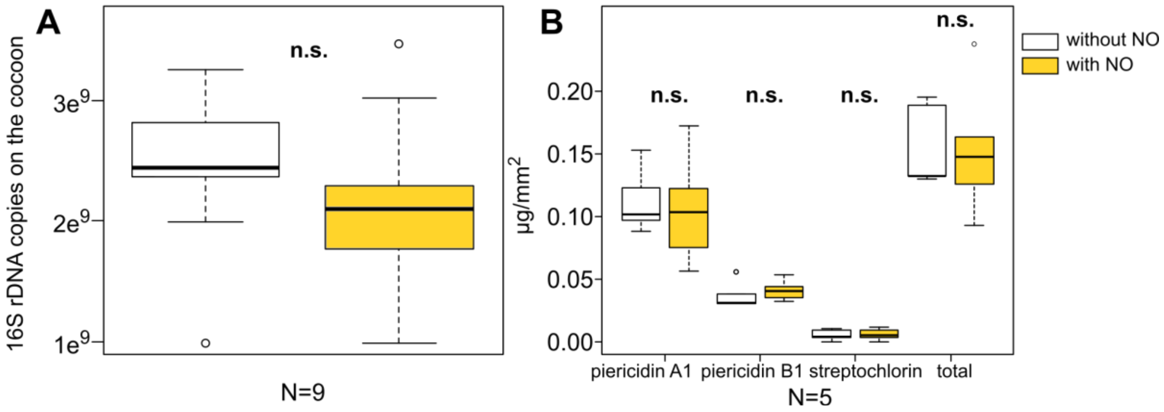

Fig. S8. Titers and antibiotic production of symbiotic *S. philanthi* on the cocoon remain unaffected by exposure to NO in the brood cell. (A) Titers of symbionts on the cocoon after exposure to NO released by the beewolf egg within the secreted AGS from beewolf brood cells, compared to titers of symbionts transiently removed from the brood cells during NO release. Titers on the cocoon surface were quantified seven days after cocoon spinning. Paired t-test, t=-0.835, df=8, p=0.428, N=9. (B) Amount of antibiotics on the cocoon surface. The same cocoons as in (A) were used. Paired t-tests, p>0.05.

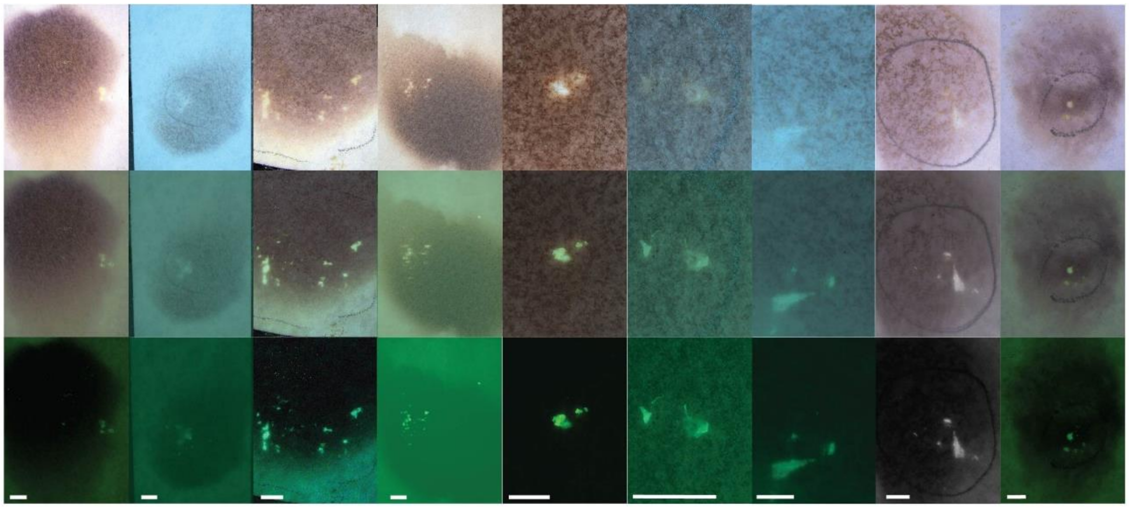

Fig. S9. The AGS prevents diffusion of NO to a filter paper containing NO indicator solution (iodine-starch solution). The figure shows the raw data to the data summarized in Figure 4D of the main manuscript. Each scale bar indicates 2 mm. (A) Light micrographs of filter paper with AGS after exposure to NO in brood cells. (B) Autofluorescence micrographs of the same areas as in (A). (C) Superimposed images.

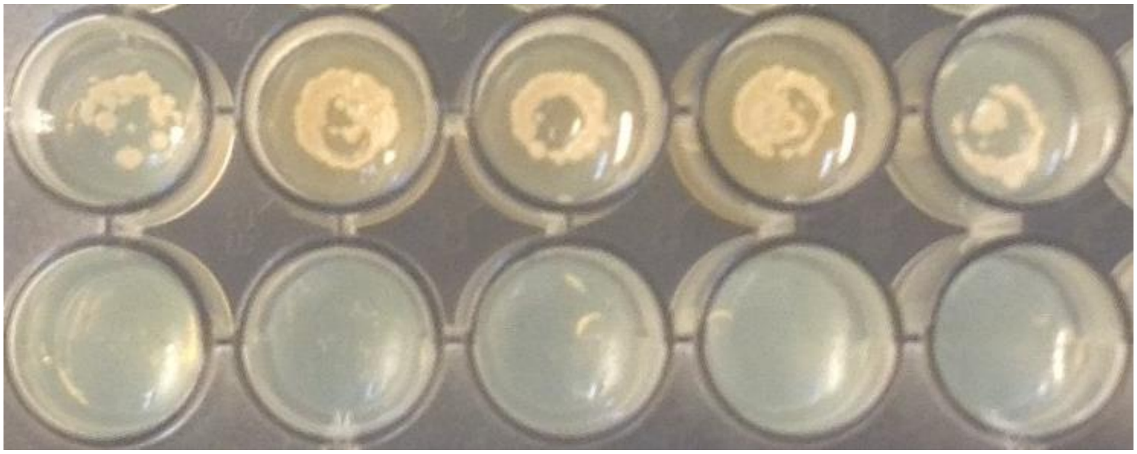

Fig. S10. Synthetic hydrocarbons protect growing *S. philanthi* cultures against lethal NO exposure in vitro. Top row: cultures covered in (Z)-9-tricosene during exposure. Bottom row: control cultures without hydrocarbons.

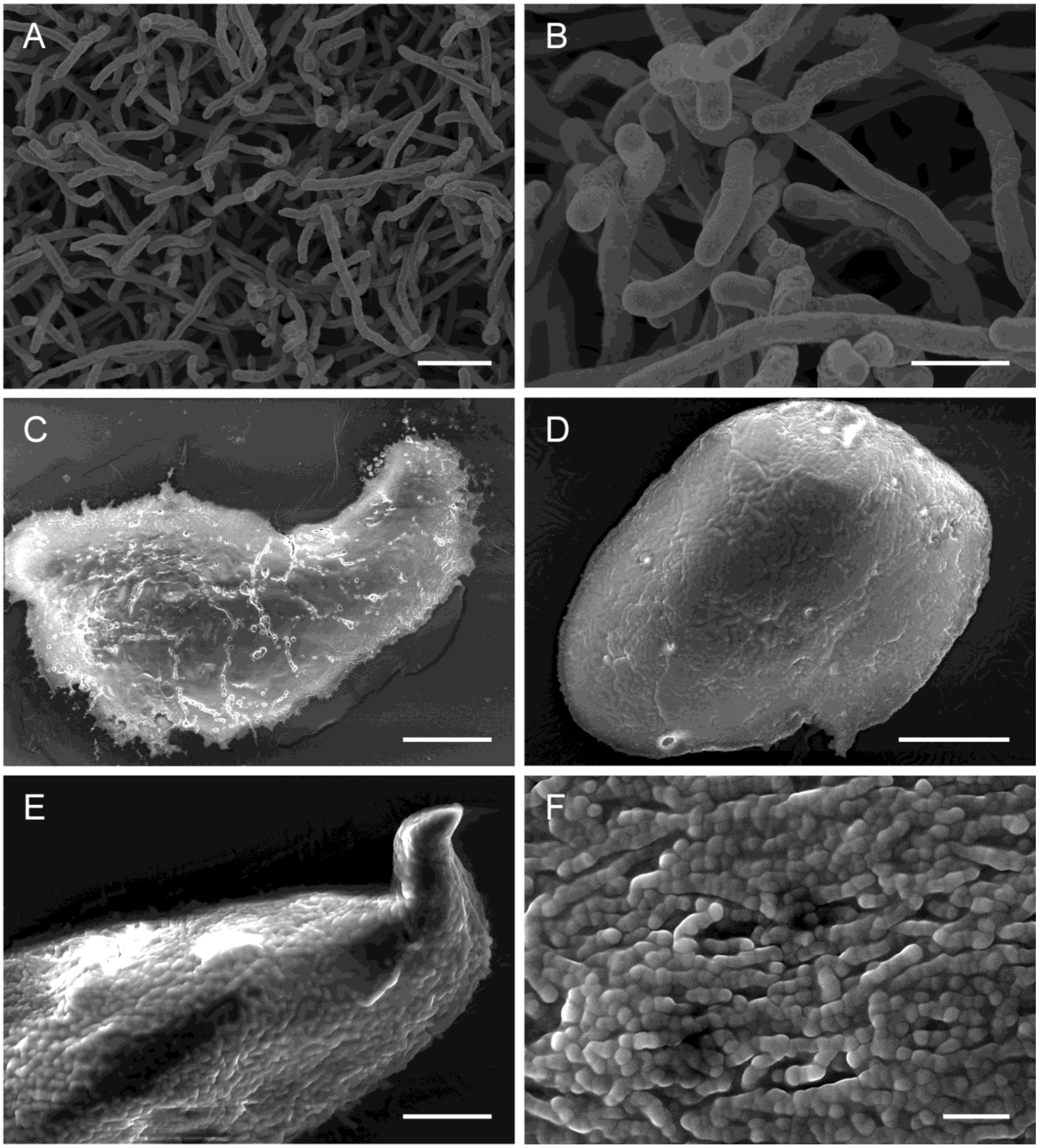

Fig. S11. Ultrastructure of *S. philanthi* cells in vitro and in the AGS. (A,B) Scanning electron micrographs (SEM) of *S. philanthi* growing in vitro in Grace's medium. (C-E) SEM micrographs of the AGS from three different beewolf brood cells. (F) Close-up of (E), showing individual *S. philanthi* cells covered by the AGS matrix that is rich in hydrocarbons. Scale bars: (A) 5 µm, (B) 2 µm, (C) 200 µm, (D) 50 µm, (E) 20 µm, (F) 5 µm.

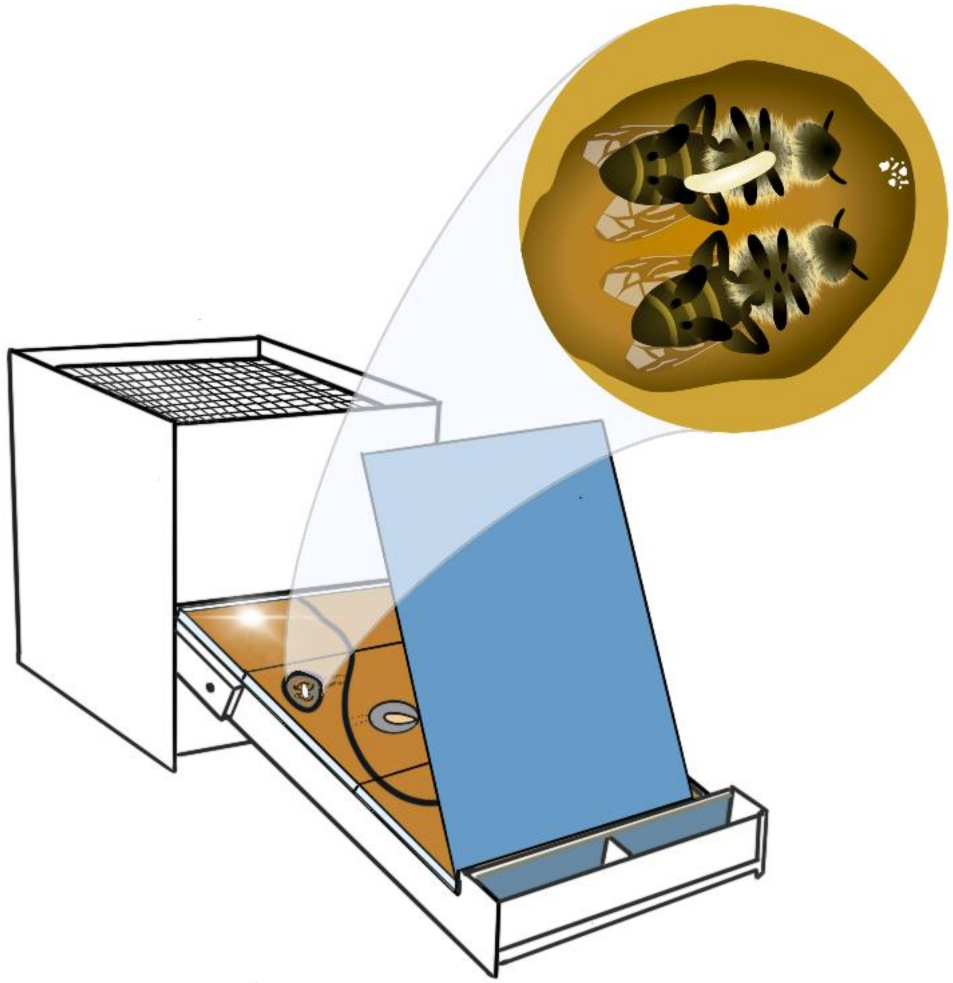

Fig. S12. Setup of a beewolf observation cage, with an exemplary brood cell (enlarged), showing a beewolf egg on the provisioned honeybees as well as the AGS on the ceiling of the brood cell.

Table S1. Number of expressed protein-coding genes in the beewolf symbiont *S. philanthi* (7930 protein-coding sequences in total) and the free-living *S. coelicolor* (7825 protein-coding sequences in total) for each treatment-time point combination of the in vitro gene expression analysis (three replicates per species, treatment and time point).

**Replicate**	***S. philanthi*** **(#)**	***S. coelicolor*** **(#)**
N_2 2h (1)	7898	7626
N_2 2h (2)	7914	7653
N_2 2h (3)	7907	7636
N_2 6h (1)	7900	7621
N_2 6h (2)	7911	7656
N2 6h (3)	7913	7624
NO 2h (1)	7902	7613
NO 2h (2)	7852	7613
NO 2h (3)	7852	7616
NO 6h (1)	7870	7586
NO 6h (2)	7827	7606
NO 6h (3**)**	7880	7648

Table S2. Number of protein-coding genes expressed by symbiotic *S. philanthi* (7930 protein-coding genes in total) for each treatment of the *in vivo* gene expression analysis.

Treatment	Replicate	# expressed genes
NO-unexposed	B	7421
	G	7313
	J	7622
	K	7515
	N	6944
NO-exposed	A	7503
	C	7617
	E	7660
	L	7526
	M	7647

Table S3. Impact of NO exposure in the brood cell on symbiont titers and antibiotic production on the cocoon. Shown are the statistical analyses of DNA copies on the cocoon and amount of the major antibiotics per cocoon area.

**Dataset**	**N**	**Shapiro-Wilk normality test of differences**	**Paired t-test**
DNA copies	9	W=0.840, p=0.058	t=-0.835, df=8, p=0.428
Piericidin A1 [µg/mm^2]	5	W=0.921, p=0.536	t=-0.691, df=4, p=0.528
Piericidin B1 [µg/mm^2]	5	W=0.944, p=0.693	t=-0.520, df=4, p=0.631
Streptochlorin [µg/mm^2]	5	W=0.861, p=0.232	t=-0.081, df=4, p=0.939
Total amount of antibiotics [µg/mm^2]	5	W=0.966, p=0.847	t=-0.179, df=4, p=0.867

Table S4. Impact of NO exposure in the brood cell on symbiont titers on the cocoon, given as DNA copies on the respective cocoons.

Individual	Brood cell	Treatment	DNA copies
1	6_1	NO	$2.096e^{9}$
1	24_1	w/o NO	$2.363e^{9}$
4	28_1	NO	$3.022e^{9}$
4	19_1	w/o NO	$2.527e^{9}$
5	19_2	NO	$1.766e^{9}$
5	10_1	w/o NO	$9.850e^{8}$
6	17_1	NO	$9.822e^{8}$
6	17_2	w/o NO	$2.813e^{9}$
8	27_4	NO	$1.281e^{9}$
8	14_3	w/o NO	$3.258e^{9}$
9	27_2	NO	$3.476e^{9}$
9	27_4	w/o NO	$2.933e^{9}$
10	15_3	NO	$2.297e^{9}$
10	15_4	w/o NO	$2.444e^{9}$
11	18_8	NO	$2.267e^{9}$
11	18_3	w/o NO	$2.441e^{9}$
11	5_7	NO	$2.086e^{9}$
11	5_9	w/o NO	$1.992e^{9}$

Table S5. Cocoon measurements, cocoon area, and antibiotics per cocoon area of brood cells in which the symbionts were or were not exposed to the NO produced by the beewolf egg. PA1 = piericidin A1, PB1 = piericidin B1, S = streptochlorin, total = total amount of antibiotics.

**Individual**	**Brood cell**	**Treatment**	**Length [mm]**	**Width [mm]**	**Area [mm^2]**	**PA1 [µg]**	**PB1 [µg]**	**S [µg]**	**total [µg]**
11	18_8	NO	15.2	4.6	69.92	8.57	2.48	0.38	11.42
11	18_3	w/o NO	14.1	4.1	57.81	7.11	3.24	0.57	10.92
11	5_7	NO	15.0	5	75.00	4.23	2.46	0.27	6.96
11	5_9	w/o NO	13.4	3.7	49.58	4.38	1.90	0.19	6.46
4	28_1	NO	16.1	4.8	77.28	5.84	3.16	0.73	9.73
4	19_1	w/o NO	20.5	6.3	129.15	13.16	3.99	0.00	17.13
6	17_1	NO	13.75	3.6	49.50	5.12	2.20	0.00	7.31
6	17_2	w/o NO	19.3	5.2	100.36	9.76	3.07	0.46	13.30
8	27_4	NO	17.0	4.5	76.50	13.16	4.09	0.90	18.15
8	14_3	w/o NO	17.35	4.85	84.15	12.88	2.65	0.92	16.44

Table S6. Protective activity of the AGS against NO diffusion in the beewolf brood cell. Given are the mean gray values of the NO-indicator filter paper bioassay.

Brood cell	Treatment	Mean gray value
1	AGS	1.755
	Filter paper NO	3.349
	Control	1.092
2	AGS	1.630
	Filter paper NO	1.990
	Control	1.021
3	AGS	1.333
	Filter paper NO	1.839
	Control	1.043
4	AGS	1.347
	Filter paper NO	1.690
	Control	1.107
5	AGS	1.226
	Filter paper NO	1.689
	Control	0.914
6	AGS	1.324
	Filter paper NO	1.926
	Control	1.032
7	AGS	1.757
	Filter paper NO	2.649
	Control	0.845
8	AGS	3.576
	Filter paper NO	3.920
	Control	0.934
10	AGS	1.151
	Filter paper NO	1.412
	Control	1.026

Table S7. Diffusion barrier effect of beewolf hydrocarbons on an iodine starch indicator solution exposed to NO. The numbers represent spectrophotometrically measured absorbance values at 540 nm of differently treated NO indicator solutions after 1h of NO exposure.

**Sample**			**OD_{540}**			
Beewolf CHC extract	0.090	0.102	0.058	0.063	0.053	0.053
Indicator solution + hexane	1.764	1.672	1.716	1.840	1.706	1.820
Untreated indicator solution	0.053	0.057	0.046	0.048	0.053	0.053

SI References

1. Poole R, Nitric oxide and nitrosative stress tolerance in bacteria. *Biochem. Soc. Trans.* **33,** 176-180 (2005).
2. Valko M, Jomova K, Rhodes CJ, Kuča K, Musílek K, Redox-and non-redox-metal-induced formation of free radicals and their role in human disease. *Arch. Toxicol.* **90,** 1-37 (2016).
3. Cassat JE, Skaar EP, Iron in infection and immunity. *Cell Host Microbe* **13,** 509-519 (2013).
4. Jackett P, Aber V, Lowrie D, Virulence and resistance to superoxide, low pH and hydrogen peroxide among strains of *Mycobacterium tuberculosis*. *Microbiology* **104,** 37-45 (1978).
5. Piddington DL *et al.*, Cu, Zn superoxide dismutase of *Mycobacterium tuberculosis* contributes to survival in activated macrophages that are generating an oxidative burst. *Infect. Immun.* **69,** 4980-4987 (2001).
6. Manca C, Paul S, Barry III CE, Freedman VH, Kaplan G, *Mycobacterium tuberculosis* catalase and peroxidase activities and resistance to oxidative killing in human monocytes in vitro. *Infect. Immun.* **67,** 74-79 (1999).
7. Ng VH, Cox JS, Sousa AO, MacMicking JD, McKinney JD, Role of KatG catalase-peroxidase in mycobacterial pathogenesis: countering the phagocyte oxidative burst. *Mol. Microbiol.* **52,** 1291-1302 (2004).
8. Reniere ML, Reduce, induce, thrive: bacterial redox sensing during pathogenesis. *J. Bacteriol.* **200,** e00128-00118 (2018).
9. Fang FC, Perspectives series: host/pathogen interactions. Mechanisms of nitric oxide-related antimicrobial activity. *J. Clin. Investig.* **99,** 2818-2825 (1997).
10. Wurtmann EJ, Wolin SL, RNA under attack: cellular handling of RNA damage. *Crit. Rev. Biochem. Mol. Biol.* **44,** 34-49 (2009).
11. Grant R, Filman D, Finkel S, Kolter R, Hogle J, The crystal structure of Dps, a ferritin homolog that binds and protects DNA. *Nat. Struct. Biol.* **5,** 294-303 (1998).
12. Reichmann D, Voth W, Jakob U, Maintaining a healthy proteome during oxidative stress. *Mol. Cell* **69,** 203-213 (2018).
